# Mitochondrial cardiolipin remodeling facilitates efficient myoblast differentiation

**DOI:** 10.1016/j.jlr.2025.100909

**Published:** 2025-09-23

**Authors:** Yohsuke Ohba, Chinami A. Fujiwara, Makoto Arita

**Affiliations:** 1 Division of Physiological Chemistry and Metabolism, Graduate School of Pharmaceutical Sciences, Keio University, Tokyo, Japan; 2 Laboratory for Metabolomics, RIKEN Center for Integrative Medical Sciences (IMS), Yokohama, Kanagawa, Japan; 3 Cellular and Molecular Epigenetics Laboratory, Graduate School of Medical Life Science, Yokohama-City University, Yokohama, Kanagawa, Japan; 4 Human Biology-Microbiome-Quantum Research Center (WPI-Bio2Q), Keio University, Tokyo, Japan

**Keywords:** lipidomics, mitochondria, muscle, phospholipid, cell signaling

## Abstract

During myoblast differentiation, mitochondria undergo dynamic changes in their morphology and function. Although the mitochondrial membrane lipid environment is closely related to mitochondrial integrity, how mitochondrial lipid composition changes during myoblast differentiation and whether it is involved in efficient differentiation remains unclear. In this study, we applied LC-MS/MS-based untargeted lipidomics to the mitochondria isolated from C2C12 murine myoblasts and found that the proportion of linoleic acid (C18:2)-containing cardiolipin (CL) increased during the early stages of differentiation. In parallel, the expression of tafazzin, a mitochondrial CL remodeling enzyme, increased in line with myoblast differentiation. Notably, the increase in C18:2-containing CL was not suppressed by the knockdown of *MyoD* (myoblast determination protein 1), a master transcription factor for myoblast differentiation. In contrast, the inhibition of CL biosynthesis and remodeling significantly suppressed differentiation progression, which was partially rescued by exogenous supplementation with C18:2. Similar trends in CL remodeling were observed when primary stem cells isolated from mouse skeletal muscle differentiated into myotubes. These results demonstrate that mitochondrial CL remodeling at an early stage is required to promote efficient myoblast differentiation.

Skeletal muscle constitutes ∼40% of the body mass and is essential for physical movements. Its dysfunction reduces exercise capacity and adversely affects overall health (1). It also plays a critical role in energy production through glucose and fatty acid metabolism (2). Skeletal muscle integrity is maintained by the efficient regeneration of muscle fibers. This regeneration, or myogenesis, is achieved by the differentiation of myogenic satellite cells (SCs). SCs undergo dynamic metabolic reprogramming during differentiation, and impaired muscle regeneration leads to loss of muscle strength, which is commonly observed in aging or muscle-wasting diseases (3).

Mitochondria are surrounded by inner and outer membranes, which contribute to diverse cellular functions, including energy production through oxidative phosphorylation (OXPHOS), calcium homeostasis, and apoptosis regulation (4). Recent studies have demonstrated that mitochondrial dysfunction affects the fate and differentiation of SCs, thereby contributing to skeletal muscle disorders (5). SCs initially contain few mitochondria and exhibit low metabolic activity, relying primarily on glycolysis for energy homeostasis. In contrast, differentiated muscle fibers have increased mitochondrial mass, fused mitochondrial networks, and robust OXPHOS capacity (6). These mature muscle fibers require functional mitochondria to accommodate the high energy demands (7).

The mitochondria possess a unique membrane lipid composition that is closely associated with their function (8). While mitochondrial membranes contain various phospholipids, only a few (phosphatidylethanolamine [PE], phosphatidylglycerol, and cardiolipin [CL]) are synthesized within the mitochondria, whereas the rest are imported from other cellular compartments. CL is a signature mitochondrial phospholipid associated with energy production and mitochondrial morphology (9). CL is synthesized from phosphatidic acid (PA) in the inner mitochondrial membrane (IMM). The newly synthesized CL, which is initially rich in saturated fatty acyl chains, undergoes remodeling to acquire unsaturated fatty acyl chains, primarily linoleic acid (C18:2) (10). Deficiency in CL remodeling leads to Barth syndrome (BTHS), an X-linked genetic disorder characterized by cardiomyopathy and skeletal myopathy (11). BTHS is caused by mutations in the tafazzin (Taz) gene, which encodes a transacylase that is essential for CL remodeling. Muscles from patients with BTHS exhibit decreased levels of unsaturated CL species, reduced total CL content, and compromised mitochondrial function (12). *Taz* KO myoblasts consistently show impaired mitochondrial function and defective myoblast differentiation (13, 14). StarD7, a mitochondrial lipid transfer protein responsible for transporting phosphatidylcholine (PC) and maintaining mitochondrial PC composition, is essential for myoblast differentiation. *StarD7* KO C2C12 cells exhibit mitochondrial respiratory defects and impaired differentiation (15). These findings highlight the importance of mitochondrial membrane homeostasis in myoblast differentiation. However, in these KO models, defects in mitochondrial lipid composition and function are present in the undifferentiated state, making it difficult to determine the specific timing at which lipids are critical for differentiation.

In this study, we applied immunoprecipitation (IP)-mediated mitochondrial isolation to C2C12 cells and conducted untargeted lipidomics to comprehensively analyze mitochondrial membrane composition during myoblast differentiation. Our results demonstrated for the first time that the enrichment of C18:2-containing CL at the early stages of differentiation is required to promote the efficient progression of myogenesis.

## Materials and Methods

### C2C12 cell culture and differentiation

Murine myoblast C2C12 cells obtained from ATCC were maintained in DMEM supplemented with l-glutamine, 10% fetal bovine serum, penicillin (100 unit/ml), and streptomycin (100 μg/ml). Cell lines were maintained at 37 °C and 5% CO_2_ and were routinely tested for mycoplasma infections. To induce myoblast differentiation, C2C12 cells at 80% confluence were switched to the differentiation medium. The differentiation medium consisted of DMEM supplemented with l-glutamine, 2% horse serum (Gibco), penicillin (100 unit/ml), and streptomycin (100 μg/ml). The differentiation medium was changed every 24 h. C2C12 cells were collected before differentiation (day 0) and after differentiation induction (days 1, 3, 5, and 7). For oligomycin or fatty acid treatment, C2C12 cells were treated with 10 nM oligomycin A (Sigma), 10 μM oleic acid (Sigma: O3008), or 10 μM linoleic acid (Sigma: L9530) while differentiating. For Torin 1 treatment, undifferentiated C2C12 cells were treated with 250 nM Torin 1 (Cell Signaling Technology) for 24 h.

### Generation of C2C12 cells with HA/Myc-tagged mitochondria or HA-tagged lysosomes

Generation of Mito-tag (3xHA-EGFP-OMP25 or 3xMyc-EGFP-OMP25) or Lyso-tag (TMEM192-3xHA) stably expressing C2C12 cells was performed as described previously (16, 17). Briefly, Plat-A cells (Cell Biolabs, Inc) were transiently transfected with pMXs-3xHA-EGFP-OMP25 (Addgene #83356) or pMXs-3xMyc-EGFP-OMP25 (Addgene #83355), or human embryonic kidney 293T cells (ATCC) were transiently transfected with pLJC5-Tmem192-3xHA (Addgene #102930) using Lenti-X packaging single shots (VSV-G) (Takara). Virus-containing culture media were collected 2 days after transfection. For viral infection, C2C12 cells were exposed to virus media containing 4 μg/ml polybrene. Blasticidin S (10 μg/ml) selection for Mito-tagged cells or puromycin (2 μg/ml) selection for Lyso-tagged cells was initiated 2 days after infection.

### Mitochondria or lysosome isolation from Mito-tag- or Lyso-tag-expressing C2C12 cells

IP of mitochondria or lysosomes from Mito-tag- or Lyso-tag-expressing C2C12 cells was performed according to a previously described protocol (16, 17). Briefly, C2C12 cells (∼30 million cells) were scraped with PBS, and collected cell pellets were resuspended with 500 μl KPBS (pH 7.25) (136 mM KCl and 10 mM KH_2_PO_4_). Cells were homogenized using a 27 G syringe (Becton Dickinson) (15 strokes) and centrifugated at 600 *g* for 5 min. Supernatants were mixed with 100 μl of anti-HA magnetic beads (Thermo Fisher Scientific) and incubated on a rotator for 10 min at 4°C. Beads were washed four times with KPBS, and 10% of mitochondria- or lysosome-captured HA magnetic beads from each condition were kept for the validation of organelle isolation by immunoblotting, and the rest of the HA magnetic beads (90% of total) were kept for lipidomics.

### Microsomal fractionation

Microsomal fractionation was performed according to a previously described protocol (18). Collected C2C12 cells were homogenized using a 27 G syringe (Becton Dickinson) (15 strokes) in the presence of homogenization buffer (220 mM mannitol, 70 mM sucrose, 5 mM Hepes/KOH [pH 7.4], and 1 mM EGTA) and centrifugated at 600 *g* for 5 min. The resulting supernatant was further centrifuged at 8,000 *g* for 10 min at 4 °C, and the supernatant was collected. The ultracentrifugation (100,000 *g*, 30 min, 4°C) was performed, and the resulting pellet was used as a microsome fraction.

### siRNA transfection

Transfection of C2C12 cells with siRNAs was performed using Lipofectamine RNAiMAX Transfection Reagent (Thermo Fisher Scientific) according to the manufacturer’s protocol. The siRNAs (Thermo Fisher Scientific) used in this study are listed in supplemental Table S1. An siRNA without any mouse target was used as a control (Silencer^TM^ Select Negative Control No. 1 siRNA [Thermo Fisher Scientific]). C2C12 cells were transfected twice after plating and induction of myoblast differentiation. The timing of the siRNA transfection is shown in supplemental Fig. S4A.

### Quantitative real-time PCR

Total RNA was extracted from cells using ISOGEN II (NIPPON GENE), and complementary DNA was synthesized using ReverTra Ace quantitative PCR (qPCR) RT Master Mix (TOYOBO). For real-time qPCR, the synthesized complementary DNA products were reacted with THUNDERBIRD Next SYBR qPCR Mix (TOYOBO) using the CFX Connect Real-Time System (Bio-Rad Laboratories). ACTB was used as a control to normalize the mRNA expression levels. Primers used for amplification are listed in supplemental Table S2.

### Immunoblotting

Cells were washed with ice-cold PBS and lysed with RIPA buffer (50 mM Tris-HCl [pH 7.4], 150 mM NaCl, 1 mM EDTA, 1% [v/v] Triton X-100, 0.1% [w/v] SDS, and 0.5% [w/v] sodium deoxycholate) containing cOmplete EDTA free (Roche) for 30 min. After centrifugation at 15,000 rpm for 10 min at 4°C, supernatant fractions were collected and analyzed using SDS-PAGE and immunoblotting. Protein concentration from cell extract was determined using the Pierce BCA Protein Assay Kit (Thermo Fisher Scientific). Primary antibodies used in this study are listed in supplemental Table S3.

### Immunofluorescence

C2C12 cells grown on glass coverslips were fixed with 4% (v/v) paraformaldehyde in PBS for 15 min, permeabilized with 0.1% (v/v) Triton X-100 for 10 min, and incubated with primary antibodies in PBS containing 3% (w/v) bovine serum albumin. After washing, the cells were incubated with Alexa Fluor-conjugated secondary antibody. After washing, coverslips were mounted onto slides using ProLong Gold (Thermo Fisher Scientific) and imaged using a confocal fluorescence microscope (FV3000; Olympus). Primary antibodies used in this study are listed in supplemental Table S3.

### Respiration measurements

Oxygen consumption rate (OCR) levels were measured using a Seahorse Extracellular Flux Analyzer XFe96 (Agilent). C2C12 cells (2 × 10^4^) were plated in each well. The assay medium consisted of DMEM supplemented with glucose (25 mM) and glutamine (2 mM). Cells were washed twice with assay media and incubated for 1 h in a 37°C non-CO_2_ incubator before starting the assay. The basal OCR was normalized to the amount of protein per well using the Bradford assay (Bio-Rad).

### Isolation of skeletal muscle stem cells and induction of differentiation

Muscle stem cell (MuSC) isolation from mouse skeletal muscle tissues and differentiation was induced according to a previously described protocol (19). Isolated SCs were cultured in Matrigel-coated dishes in DMEM supplemented with 30% FBS, 1% chick embryo extract (US Biological), basic fibroblast growth factor (10 ng/ml; Wako), and 1% penicillin-streptomycin (Gibco). MuSC isolation was validated based on cell morphology (microscopic observation) and Pax7 expression (immunoblotting). Myogenic differentiation was induced in DMEM supplemented with 5% horse serum (Gibco) and 1% penicillin-streptomycin for up to 5 days. This experiment was approved by and performed in accordance with the Guidelines for Animal Experimentation of the Animal Use Committee at Keio University.

### Untargeted lipidomics

Total lipids were extracted using a single-phase extraction method with a mixed solvent (MeOH:CHCl_3_:H_2_O, 2:1:0.2, v/v/v) as previously described (18). Briefly, 1 million cells, 20 μg protein of microsome fraction, 90% of mitochondria- or lysosome-captured HA magnetic beads, or 20 μl of culture media were mixed with 200 μl of MeOH containing 5 μl of EquiSPLASH (Avanti Polar Lipids) and incubated for 1 h at room temperature. CHCl_3_ (100 μl) was added, and the samples were further incubated for 1 h. Finally, 20 μl of Milli-Q water was added and incubated for 10 min (except for extracting lipids from media). After extraction, the samples were centrifuged at 2,000 *g* for 10 min, and the supernatants were collected for lipidomic analysis. Extracted lipids were analyzed using an LC-QTOF/MS system (LCMS-9030; Shimadzu) operated in data-dependent acquisition mode, as described previously (18). LC separation was performed using an ACQUITY UPLC Peptide BEH C18 (2.1 mm × 50 mm; Waters) with mobile phase A (methanol:acetonitrile:water = 1:1:3, v/v/v, containing 5 mM ammonium acetate and 10 nM EDTA) and B (2-propanol containing 5 mM ammonium acetate and 10 nM EDTA). The flow rate was 0.3 ml/min, and the column was kept at 45°C. LC gradient consisted of solvent A for 0.5 min, then linearly converting to solvent (A:B = 60:40) for 4 min, linearly converting to solvent (A:B = 36:64) for 2.5 min and holding for 4.5 min, then linearly converting to solvent (A:B = 17.5/82.5) for 0.5 min, linearly converting to solvent (A:B = 15:85) for 6.5 min, and linearly converting to solvent (A:B = 5:95) for 1 min followed by returning to solvent A and holding for 5 min. The parameters for data-dependent acquisition were identical to those reported previously (18); MS1 and MS2 start-end time: 0.5–18.5 min, MS1 and MS2 mass ranges: *m/z* 70–1750, collision energy: 35 eV, collision energy spread: 20 eV, MS1 event time: 250 ms, MS2 event time: 66 ms, interface voltage: 4.00 kV (+)/−3.00 kV (−), number of dependent events: 15, collision-induced dissociation gas: argon, 230 kPa, ionization mode: ESI, nebulizer gas: 2.0 l/min, heating gas: 20.0 l/min (+)/10.0 l/min (−), interface temperature: 100°C (+)/300°C (−), drying gas: 10.0 l/min, desolvation line temperature: 250°C (+)/300°C (−), and heat block temperature: 400°C. The obtained data were processed using MS-DIAL 5 (20). The analysis parameters were identical to those reported previously (18). Concentrations of EquiSPLASH-containing lipid classes were calculated from the peak height of the precursor ion of deuterated standards of each lipid in EquiSPLASH. The concentrations of other lipid classes were calculated from the peak height of the precursor ions of the deuterated standards of appropriate lipids in EquiSPLASH. Detailed information is described in supplemental data.

### Statistical analyses

Statistical significance was assessed using a two-tailed *t*-test or one-way ANOVA as indicated. All reported numbers refer to biological replicates. Statistical significance was set at *P* < 0.05.

## Results

### Comprehensive analysis of mitochondrial membrane lipids during myoblast differentiation

**Fig. 1:**
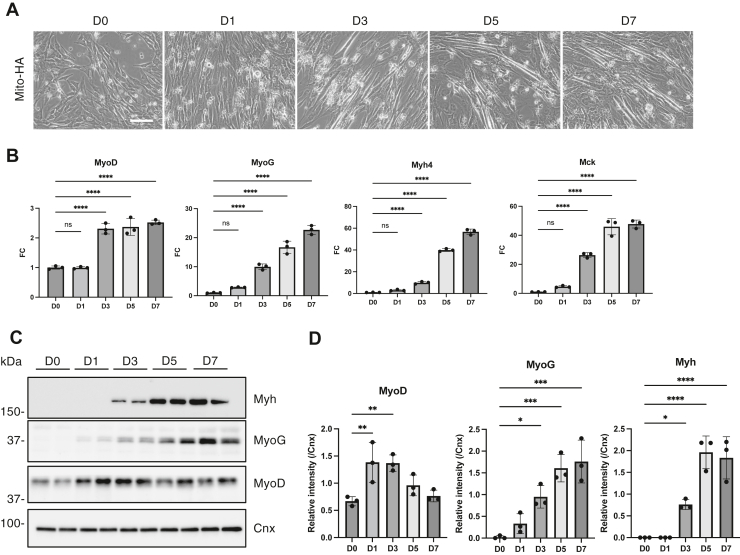
Myoblast differentiation of Mito-HA-expressing C2C12 cells. A: Phase-contrast images of Mito-HA-expressing C2C12 cells cultured in the differentiation medium for 7 days. The scale bar represents 10 μm. B: The mRNA levels of *MyoD*, *MyoG*, *Myh4*, and *Mck* during C2C12 cell differentiation were quantified by qRT-PCR (n = 3). C and D: Immunoblot analysis of myoblast differentiation markers (MyoD, MyoG, and Myh) during C2C12 cell differentiation (C). The indicated antibodies were used, as appropriate. The protein expression levels were quantified (D) (n = 3). Mean ± SD; ∗*P* < 0.05; ∗∗*P* < 0.01; ∗∗∗*P* < 0.001; ∗∗∗∗*P* < 0.0001; ns, not significant, one-way ANOVA with Dunnett’s multiple comparisons test.

To analyze mitochondrial lipids during myoblast differentiation, we used mitochondrial immunoprecipitation (Mito-IP) in the murine myoblast cell line C2C12 to isolate mitochondria (16). This approach enables the rapid isolation of mitochondria compared with conventional methods. First, we generated either Mito-HA (3xHA-EGFP-OMP25)- or Mito-Myc (3xMyc-EGFP-OMP25)-expressing C2C12 cells. The tags were correctly expressed and colocalized with the mitochondrial outer membrane protein TOMM20 in C2C12 cells (supplemental Fig. S1A, B). Mito-HA-expressing C2C12 cells retained their ability to differentiate into myotubes with an elongated tubular morphology (Fig. 1A). The expression of typical muscle differentiation markers, including protein levels of myosin heavy chain (Myh), myoblast determination protein 1 (MyoD), and myogenin (MyoG) and mRNA levels of *MyoD*, *MyoG*, *Myh4*, and muscle creatine kinase (*Mck*), increased during differentiation in Mito-HA-expressing C2C12 cells (Fig. 1B–D). Mitochondrial biogenesis is upregulated during myoblast differentiation (21), and we observed an increased expression of OXPHOS components using both an OXPHOS antibody cocktail and Sdha (succinate dehydrogenase complex flavoprotein subunit A)-specific antibodies (supplemental Fig. S1D, E), indicating the occurrence of dynamic changes in mitochondrial function during differentiation.

**Fig. 2:**
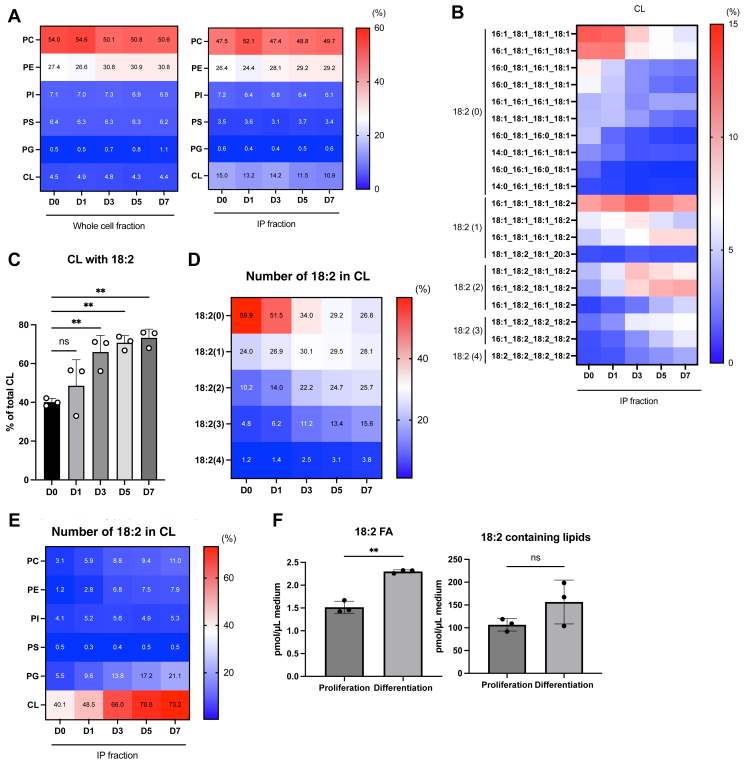
Comprehensive analysis of mitochondrial membrane phospholipids during myoblast differentiation of Mito-HA C2C12 cells. A: The proportion of each phospholipid in either the whole cell fraction or the IP fraction is shown as a heatmap with mean values (n = 3). B: The proportion of CL in the IP fraction for each fatty acid composition is shown as a heatmap with the mean value (n = 3). The number of linoleic acid moieties (18:2) is shown on the left side of the heatmap. C: The proportion of CL with 18:2 (n = 3). D: The proportion of CL with each number of 18:2 is shown as a heatmap with mean values (n = 3). E: The proportion of each phospholipid containing 18:2 is shown as a heat map with a mean value (n = 3). F: The amount of C18:2- (18:2 FA) and C18:2-containing lipids in the proliferation or differentiation media (n = 3). Mean ± SD; ∗∗*P* < 0.01; ns, not significant, one-way ANOVA with Dunnett’s multiple comparisons test (C) or unpaired Student’s *t*-test (F).

Next, we analyzed the cellular and mitochondrial membrane phospholipids using LC-QTOF-MS-based untargeted lipidomics (22). Mito-HA-expressing C2C12 cells were collected at multiple time points before and after inducing differentiation, and mitochondria were isolated by IP. The enrichment of mitochondrial proteins (Sdha, Vdac1, and Tomm20) and exclusion of proteins from the endoplasmic reticulum (calreticulin), lysosomes (Lamp1 [lysosome-associated membrane protein]), and cytosol (Gapdh) confirmed the specificity of mitochondrial isolation (supplemental Fig. S2A). Mito-Myc-expressing undifferentiated C2C12 cells served as negative controls for Mito-IP (supplemental Fig. S2A). Consistent with the immunoblotting results, lipidomic analysis supported mitochondrial enrichment in the IP fractions. The proportion of CL was higher in Mito-IP fractions than in whole-cell lysates (4.5% in whole-cell vs. 15.0% in Mito-IP on day 0) (Fig. 2A). Significant alterations were observed in the fatty acid composition of each major phospholipid class in the Mito-IP fractions during differentiation, whereas the relative abundance of major phospholipids remained unchanged across time points in both whole-cell and Mito-IP fractions (Fig. 2A, B and supplemental Fig. S2B). For instance, in PE, molecules containing arachidonic acid (C20:4) or docosahexaenoic acid (C22:6), such as PE 18:0_20:4, PE 18:1_20:4, and PE 18:0_22:6, decreased, whereas those containing oleic acid (C18:1), including PE 16:0_18:1, PE 18:0_18:1, PE 18:1_18:1, PE 16:1_18:1, and PE_18:1_18:2, increased on day 1 after induction (supplemental Fig. S2B). Similarly, in phosphatidylinositol (PI), C20:4-containing species (e.g., PI 18:0_20:4 and PI 18:1_20:4) decreased (supplemental Fig. S2B). Among the major phospholipid classes, a notable change was observed in CL; the proportion of C18:2-containing CL species significantly increased at the early stage of differentiation (by day 3) and remained elevated thereafter (Fig. 2B–E). Since the culture media for proliferation and differentiation were different, we compared the lipid composition of those culture media and found that the amount of C18:2- and C18:2-containing lipids was relatively high in differentiation media (1.51-fold and 1.47-fold, respectively) (Fig. 2F). Among the C18:2-containing lipid species in cells, all classes except phosphatidylserine showed a slight increase, which appears to be explained by the increased C18:2 content in differentiation media (Fig. 2E). To further determine the unique lipid remodeling locally in mitochondria, lysosome and microsome fractions were isolated from C2C12 cells (Fig. S3B and S3C). Lysosome fraction was enriched from Lyso-tag-expressing C2C12 cells (Fig. S3A and S3B). Similar to mitochondria, the relative abundance of major phospholipids in lysosome and microsome fractions remained unchanged (supplemental Fig. S3D). In lysosomes, the fatty acid composition of bis(monoacylglycero)phosphate, a lysosome-enriched phospholipid class, remained unchanged (supplemental Fig. S3E). Moreover, the transition of the fatty acid composition of each major phospholipid class observed in those fractions was similar to that of mitochondria (supplemental Figs. S2B and S3E). For instance, in PE, molecules containing C20:4 decreased, whereas those containing C18:1 increased (supplemental Fig. S3E). Also, in PI, C20:4-containing species decreased (supplemental Fig. S3E). From these comprehensive organelle lipidomics during myoblast differentiation, CL remodeling appeared to represent the locally characteristic feature in mitochondria.

### Mitochondrial CL remodeling was not a consequence of myoblast differentiation

**Fig. 3:**
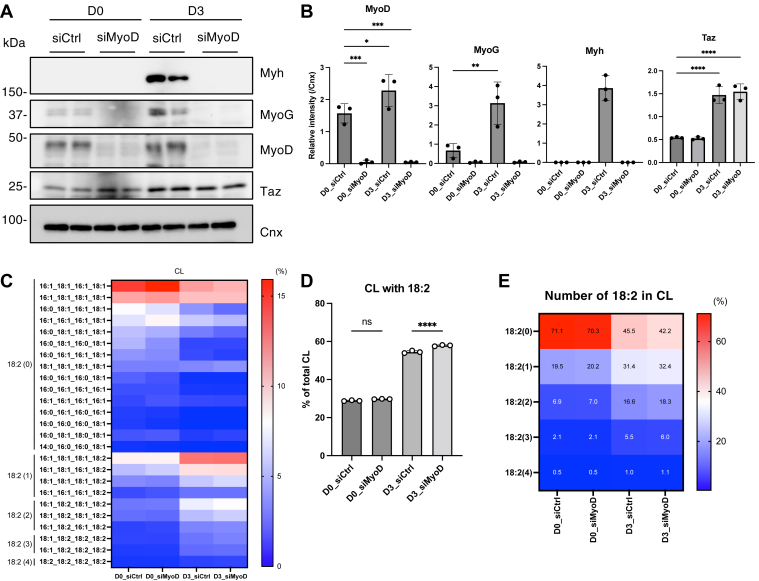
Inhibition of myoblast differentiation does not affect the transition of fatty acid composition in CL. A and B: Immunoblot analysis of indicated proteins during myoblast differentiation of either siCtrl- or siMyoD-treated C2C12 cells (A). The protein expression levels were quantified (B) (n = 3). C: The proportion of CL with each fatty acid composition is shown as a heatmap with the mean value (n = 3). The number18:2 is shown on the left side of the heatmap. D: The proportion of CL with 18:2 (n = 3). E: The proportion of CL with each number of 18:2 is shown as a heatmap with mean values (n = 3). Mean ± SD; ∗*P* < 0.05; ∗∗∗*P* < 0.001; ∗∗∗∗*P* < 0.0001; ns, not significant, one-way ANOVA with Dunnett’s multiple comparisons test (B) or Sidak’s multiple comparisons test (D).

To determine whether the increase in C18:2-containing CL was a consequence of myoblast differentiation, we knocked down *MyoD*, a muscle-specific transcription factor and key regulator of myoblast differentiation (23), using siRNA 2 days before initiating differentiation (supplemental Fig. S4A). C2C12 cells treated with MyoD-targeting siRNA (siMyoD) exhibited over a 90% reduction in both MyoD mRNA and protein expression at the time of differentiation induction, and this suppression was maintained for up to 3 days thereafter (Fig. 3A, B and supplemental Fig. S4B). The induction of myogenic markers was also significantly suppressed in siMyoD-treated cells compared with that in control siRNA-treated cells (Fig. 3A, B and supplemental Fig. S4B). Despite the complete inhibition of myoblast differentiation, induction of C18:2-containing CL was observed in siMyoD-treated cells (Fig. 3C–E). These results demonstrate that mitochondrial CL remodeling is not a consequence of myoblast differentiation.

### Inhibition of CL synthesis and remodeling suppressed myoblast differentiation

**Fig. 4:**
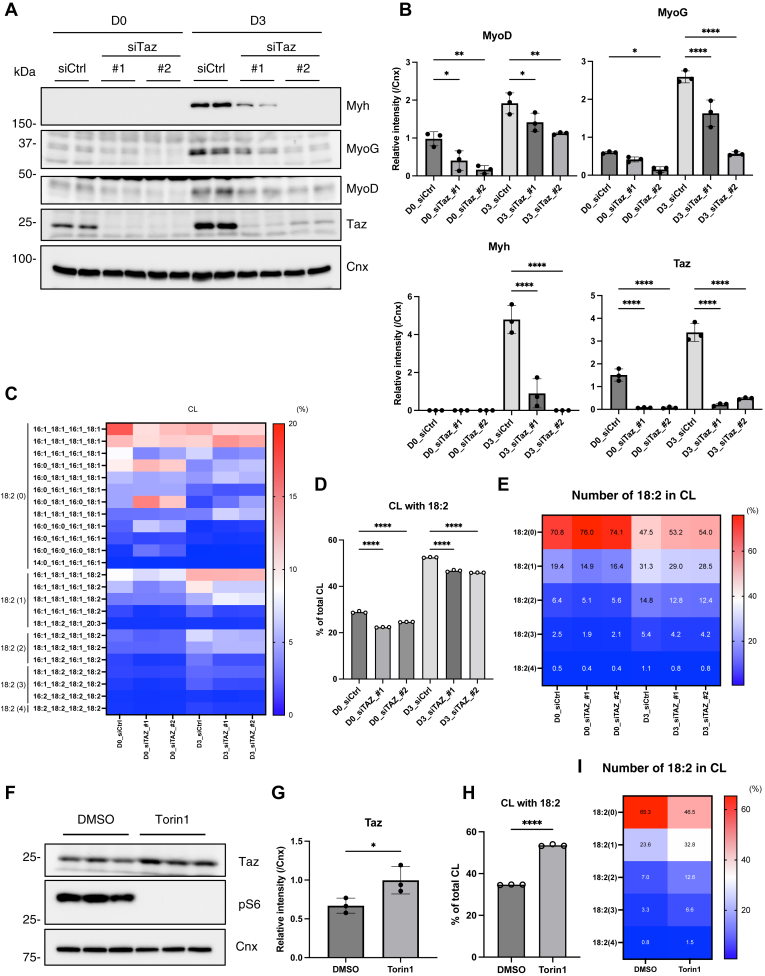
The inhibition of CL remodeling enzyme Taz suppressed the increase of CL with linoleic acid and the progress of myoblast differentiation. A and B: Immunoblot analysis of indicated proteins during myoblast differentiation of either siCtrl- or siTaz- (#1 or #2) treated C2C12 cells (A). The protein expression levels were quantified (B) (n = 3). C: The proportion of CL with each fatty acid composition is shown as a heatmap with the mean value (n = 3). The number 18:2 is shown on the left side of the heatmap. D: The proportion of CL with 18:2 (n = 3). E: The proportion of CL with each number of 18:2 is shown as a heatmap with mean values (n = 3). F and G: C2C12 cells were treated with 250 nM Torin 1 for 24 h and performed immunoblot analysis with indicated proteins (F). Taz expression levels were quantified (G) (n = 3). H: The proportion of CL with 18:2 (n = 3). I: The proportion of CL with each number of 18:2 is shown as a heatmap with mean values (n = 3). Mean ± SD; ∗*P* < 0.05; ∗∗*P* < 0.01; ∗∗∗∗*P* < 0.0001; ns, not significant, one-way ANOVA with Sidak’s multiple comparisons test (B, D) or unpaired Student’s *t*-test (G, H).

Given that C18:2-containing CL is important for mitochondrial function (24, 25) and that the mitochondrial architecture undergoes dynamic changes during myoblast differentiation (26), we hypothesized that an increase in C18:2-containing CL occurs in advance to support the myoblast differentiation process. Taz is a mitochondrial transacylase involved in CL maturation via fatty acid remodeling. Taz is known to preferentially transfer linoleic acid from PC to monolysocardiolipin (MLCL) (27, 28, 29, 30). Upon siRNA-mediated knockdown of *Taz* at the onset of differentiation, the increase in C18:2-containing CL was significantly suppressed in the early stage of differentiation (Fig. 4C–E). Under these conditions, the expression of myoblast differentiation markers was also significantly reduced (Fig. 4A, B and supplemental Fig. S5A). Notably, *Taz* knockdown did not significantly impair mitochondrial respiratory function, as evaluated by OCR measurements at the induction stage of differentiation (supplemental Fig. S5B). These results suggested that the accumulation of C18:2-containing CL at an early stage of differentiation is important for the progression of myoblast differentiation. Similarly, temporal induction of Taz expression was observed at an early stage of differentiation (Fig. 4A, B, supplemental Figs. S1D, E, and S5A), which may contribute to temporal mitochondrial CL remodeling to facilitate efficient myogenesis. To further examine the regulatory mechanism for Taz expression, we determined the potential involvement of mammalian target of rapamycin (mTOR), a protein kinase, which maintains cellular metabolism, proliferation, and homeostasis (31). mTOR signaling is reported both as negative and positive regulators of myogenesis, indicating that mTOR has a biphasic role in myoblast differentiation (32, 33, 34, 35). We found that mTOR signaling was inhibited in the early stage of myoblast differentiation, at least up to 3 days after differentiation induction, which was inversely correlated with Taz protein expression (supplemental Fig. S1D). When undifferentiated C2C12 cells were treated with mTOR inhibitor Torin 1, Taz protein expression was increased significantly (Fig. 4F, G). Along with this, C18:2-containing CL was increased by Torin 1 treatment in undifferentiated C2C12 cells (Fig. 4H, I). These results indicate that mTOR signaling is involved in the regulation of Taz protein and C18:2-containing CL levels in mitochondria.

**Fig. 5:**
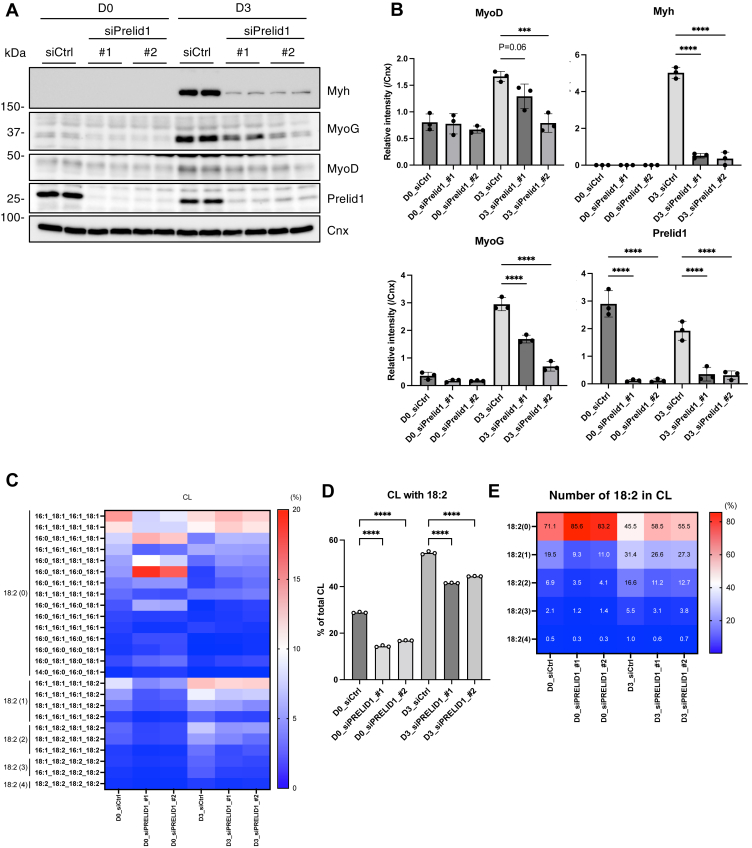
The inhibition of PRELID1, a lipid transfer protein for CL synthesis, suppressed the increase of CL with linoleic acid and the progress of myoblast differentiation. A and B: Immunoblot analysis of indicated proteins during myoblast differentiation of either siCtrl- or siPrelid1- (#1 or #2) treated C2C12 cells (A). The protein expression levels were quantified (B) (n = 3). C: The proportion of CL with each fatty acid composition is shown as a heatmap with the mean value (n = 3). The number 18:2 is shown on the left side of the heatmap. D: The proportion of CL with 18:2 (n = 3). E: The proportion of CL with each number of 18:2 is shown as a heatmap with mean values (n = 3). Mean ± SD; ∗∗∗*P* < 0.001; ∗∗∗∗*P* < 0.0001; one-way ANOVA with Sidak’s multiple comparisons test.

PRELID1 is a mitochondrial lipid transfer protein that forms a complex with TRIAP1 to transport PA across the intermembrane spaces for CL synthesis (36). In C2C12 cells, *Prelid1* siRNA effectively reduced the expression of Prelid1 (Fig. 5A and supplemental Fig. S6A). Under these conditions, the proportion of C18:2-containing CL significantly decreased (Fig. 5C–E), similar to the effect observed with Taz knockdown, although the specific relationship between PRELID1 and CL fatty acyl chain composition has not been previously reported. These findings raise the possibility that PRELID1 may preferentially transfer PA containing C18:2 or that PRELID1-mediated CL synthesis is functionally coupled with fatty acid remodeling to promote the maturation of nascent CL. siRNA-mediated knockdown of *Prelid1* at the onset of differentiation significantly suppressed myoblast differentiation, as evaluated by the expression of several differentiation markers (Fig. 5B and supplemental Fig. S6A). Together with the results of *Taz* knockdown, the temporal induction of C18:2-containing CL in the mitochondria appeared to be important for the progression of myoblast differentiation.

### Maintenance of ATP synthesis in mitochondria is required for the progression of myoblast differentiation

**Fig. 6:**
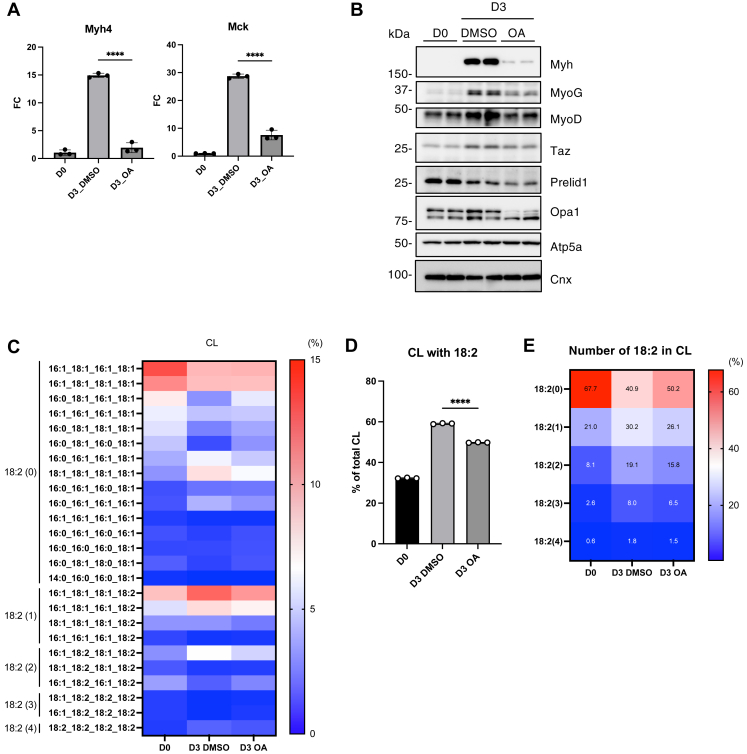
Inhibition of ATP synthase by oligomycin A (OA) affected myoblast differentiation and CL remodeling of C2C12 cells. A: The mRNA levels of Myh4 and Mck during the myoblast differentiation of OA-treated (10 nM, 3 days) C2C12 cells were quantified using qRT-PCR (n = 3). B: Immunoblot analysis of the indicated proteins during myoblast differentiation of OA-treated (10 nM, 3 days) C2C12 cells. Quantification of protein expression levels is shown in supplemental Fig. S6A. C: The proportion of CL with each fatty acid composition is shown as a heatmap with the mean value (n = 3). The number of linoleic acid molecules (18:2) is shown on the left side of the heatmap. D: The proportion of CL with 18:2 (n = 3). E: The proportion of CL with each number of 18:2 is shown as a heatmap with mean values (n = 3). Mean ± SD; ∗∗∗∗*P* < 0.0001; one-way ANOVA with Sidak’s multiple comparisons test.

Since C18:2-containing CL is important for maintaining mitochondrial function (24, 25), we hypothesized that an increase in C18:2-containing CL may support mitochondrial activity to meet the energy demands during myoblast differentiation. To test whether sustained mitochondrial function is required for differentiation, we treated C2C12 cells with a low dose (10 nM) of the ATP synthase inhibitor, oligomycin A, during the induction of myoblast differentiation. In response to oligomycin A-induced mitochondrial stress, OPA1 is cleaved by the mitochondrial protease OMA1 (37). We confirmed the Opa1 cleavage under our treatment conditions (Fig. 6B and supplemental Fig. S7A). Under these conditions, myoblast differentiation was significantly inhibited (Fig. 6A, B and supplemental Fig. S7A). These results suggest that mitochondrial ATP synthesis is necessary for myoblast differentiation. Interestingly, oligomycin A treatment significantly suppressed the increase in C18:2-containing CL (Fig. 6C–E). These results suggest that mitochondrial function is not only required for energy production but also plays a role in promoting CL remodeling.

### Linoleic acid supplementation partially restored impaired myoblast differentiation in Taz-depleted C2C12 cells

**Fig. 7:**
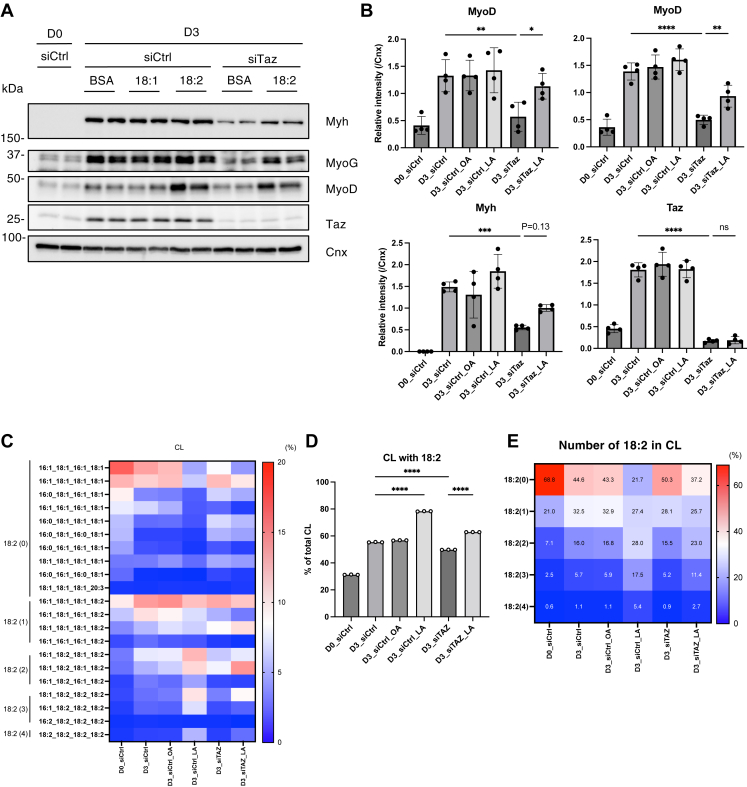
Linoleic acid supplementation partially rescued the defect of myoblast differentiation in siTaz-treated C2C12 cells. A and B: Immunoblot analysis of indicated proteins during myoblast differentiation of either siCtrl- or siTaz- (#1) treated C2C12 cells supplemented with oleic acid (18:1) or linoleic acid (18:2) (A). The protein expression levels were quantified (B) (n = 3). C: The proportion of CL with each fatty acid composition is shown as a heatmap with the mean value (n = 3). The number 18:2 is shown on the left side of the heatmap. D: The proportion of CL with 18:2 (n = 3). E: The proportion of CL with each number of 18:2 is shown as a heatmap with mean values (n = 3). Mean ± SD; ∗*P* < 0.05; ∗∗*P* < 0.01; ∗∗∗*P* < 0.001; ∗∗∗∗*P* < 0.0001; ns, not significant, one-way ANOVA with Sidak’s multiple comparisons test.

Next, we examined the effects of C18:2 supplementation on the induction of differentiation in C2C12 cells. When C18:2 was added to the culture medium of WT cells at the onset of differentiation, it was efficiently incorporated into the CL (Fig. 7C–E). However, C18:2 supplementation did not significantly enhance myoblast differentiation, except for a slight increase in *Myh4* mRNA expression (Fig. 7A, B and supplemental Fig. S8A). In contrast, supplementation with oleic acid (C18:1) neither resulted in its incorporation into the CL nor did it interfere with the incorporation of C18:2 and had no observable effect on differentiation (Fig. 7A–E). Notably, when C18:2 was supplemented to *Taz* siRNA-treated cells, it was still incorporated into the CL, although less efficiently compared with control siRNA-treated cells (Fig. 7C–E), and the impaired differentiation phenotype was partially but significantly rescued (Fig. 7A, B and supplemental Fig. S8A). These findings suggest that the differentiation defect in Taz-depleted cells results from reduced C18:2 levels in the mitochondrial CL. In contrast, the supplementation of *MyoD*-depleted or oligomycin A-treated cells with C18:2 did not restore cell differentiation (supplemental Fig. S8B), despite the efficient incorporation of C18:2 into CL, which was observed in both cases (supplemental Fig. S8C–H). These results indicate that an increase in C18:2-containing CL is required for proper myoblast differentiation and acts upstream of MyoD expression and mitochondrial function.

### CL with linoleic acid was increased during the differentiation of isolated mouse primary myoblasts

**Fig. 8:**
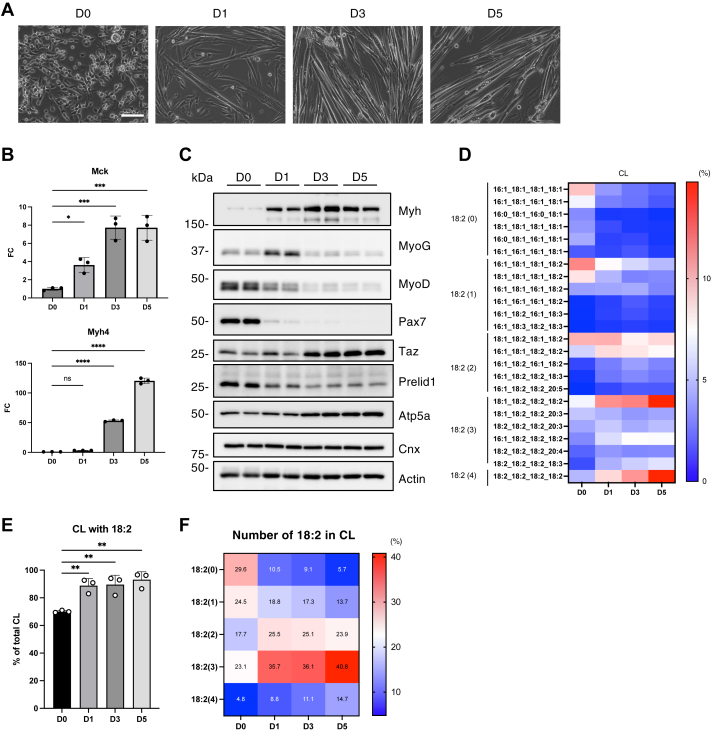
CL with linoleic acid was increased during the differentiation of isolated mouse primary myoblasts. A: Phase-contrast images of isolated mouse primary myoblasts cultured in a differentiation medium for 5 days. The scale bar represents 10 μm. B: The mRNA levels of *Myh4* and *Mck* during isolated mouse primary myoblast differentiation were quantified by qRT-PCR (n = 3). C: Immunoblot analysis of myoblast differentiation markers and mitochondrial proteins during isolated mouse primary myoblast differentiation. The indicated antibodies were used, as appropriate. Quantification of protein expression levels is shown in supplemental Fig. S8A. D: The proportion of CL with each fatty acid composition is shown as a heatmap with the mean value (n = 3). The number 18:2 is shown on the left side of the heatmap. E: The proportion of CL with 18:2 (n = 3). F: The proportion of CL with each number of 18:2 is shown as a heatmap with mean values (n = 3). Mean ± SD; ∗*P* < 0.05; ∗∗*P* < 0.01; ∗∗∗*P* < 0.001; ∗∗∗∗*P* < 0.0001; ns, not significant, one-way ANOVA with Dunnett’s multiple comparisons test.

Based on a previously reported protocol (19), we isolated skeletal MuSCs from the leg muscles of C57BL/6J mice. After inducing myoblast differentiation, we confirmed morphological changes characteristic of myotube formation, such as an elongated tubular cell shape (Fig. 8A). We also observed a decrease in the MuSC marker Pax7 and time-dependent increases in several differentiation markers and the OXPHOS component, Atp5a (Fig. 8B, C and supplemental Fig. S9A). Furthermore, consistent with the findings in C2C12 cells, Taz expression was significantly elevated, and the phosphorylation level of S6 ribosomal protein was decreased during differentiation (Fig. 8C and supplemental Fig. S9A). We then analyzed the lipid composition at different stages of differentiation and found that the proportion of C18:2-containing CL increased after differentiation was induced, despite differences in the CL acyl chain profiles between primary myoblasts and C2C12 cells (Fig. 8D–F and supplemental Fig. S9B). In undifferentiated MuSCs, C18:2-containing CL accounted for approximately 70% of the total CL, whereas undifferentiated C2C12 cells contained approximately 30% C18:2-containing CL (supplemental Fig. S9B). After differentiation, a marked increase in CL species containing two or more C18:2 chains was observed in primary cells, whereas the level of CL with one or two C18:2 chains was particularly increased in C2C12 cells (Fig. 8F and supplemental Fig. S9B). These results suggested that an increase in C18:2-containing CL is a common feature of myoblast differentiation. Furthermore, reanalysis of mice lipidomics at various life stages, which we recently reported (38), revealed that CL species without C18:2 tended to increase, whereas some CL species containing C18:2 tended to decrease with age in skeletal muscles (supplemental Fig. S9C). These changes in acyl chain composition of CL may contribute to the impairment of muscle tissue regeneration associated with aging.

## Discussion

In this study, we performed untargeted lipidomics on mitochondria, lysosomes, and microsomes isolated from C2C12 cells and identified transitions in the organelle membrane lipid environment during myogenesis. Among the several changes in organelle lipid composition, we observed an increase in CL species containing C18:2 in mitochondria following the induction of myoblast differentiation. Although the amount of C18:2 was slightly higher in differentiation media compared with proliferation media, the increased level of C18:2 was the most significant feature in CL compared with other phospholipid classes. Notably, this increase was observed even when MyoD, a critical transcription factor for myoblast differentiation, was depleted, resulting in nearly complete inhibition of differentiation. This suggests that the change in CL fatty acid composition occurs prior to, and not as a consequence of, myoblast differentiation.

How does this specific shift in CL composition occur? Nascent CL undergoes remodeling of its fatty acyl chains to produce mature CL enriched in unsaturated fatty acids such as C18:2 (30, 39). The transacylase Taz plays a central role in this remodeling process (39), and mutations in the Taz gene are a known cause of BTHS, a rare mitochondrial disorder affecting the heart and skeletal muscle (40). In the present study, we detected for the first time that *Taz* mRNA and protein levels were increased during C2C12 myogenesis, which closely paralleled an increase in C18:2-containing CL. Furthermore, the depletion of Taz shortly before the induction of differentiation significantly suppressed the increase in C18:2-containing CL, indicating that Taz upregulation accounts for this lipid transition during differentiation. Although the mechanisms regulating Taz expression and stability remain unclear, it is an unstable protein with a short half-life (41). We still observed increased Taz expression in MyoD-depleted cells, suggesting that Taz regulation does not occur downstream of MyoD. Of interest, we found that mTOR signaling was inhibited in the early stages of differentiation and was inversely correlated with Taz expression in both C2C12 cells and primary myoblasts. Furthermore, mTOR inhibitor Torin 1 treatment was sufficient to induce Taz expression and the increased level of C18:2-containing CL in C2C12 cells. These results suggest that mTOR signaling negatively regulates Taz expression, and mTOR inhibition contributes to the increase of Taz expression and CL remodeling in the early stage of differentiation. Recent studies have proposed that protein crowding on mitochondrial membranes influences Taz remodeling activity (42). Given that the expression of mitochondrial proteins, including OXPHOS components, increases during myoblast differentiation (43, 44), we speculated that the elevated expression of these proteins, along with increased Taz levels, enhances Taz activity and promotes CL remodeling. Similarly, oligomycin A, a known inhibitor of ATP synthase that disrupts the mitochondrial proteome (45), also affected the increase in CL with C18:2, possibly by altering the mitochondrial protein environment, thereby negatively affecting Taz activity. Other enzymes, such as MLCLAT1 and ALCAT1, have also been reported to catalyze the reacylation of MLCL to form CL (46), although Taz is responsible for the majority of physiological CL remodeling. The observation that a partial increase in C18:2-containing CL was still present in *Taz*-knockdown cells suggests that these alternative enzymes may also contribute to CL maturation during myoblast differentiation.

What is the physiological importance of mitochondrial CL remodeling during myogenesis? While *Taz*-KO C2C12 cells have been shown to exhibit defective differentiation and impaired mitochondrial OXPHOS, even in the undifferentiated state (14), the functional role of CL acyl chain remodeling has not been explicitly addressed. In this study, the suppression of CL remodeling by *Taz* knockdown significantly inhibited myoblast differentiation, which was partially rescued by exogenous C18:2 supplementation. Furthermore, the knockdown of *Prelid1* also suppressed both the increase in C18:2-containing CL and myoblast differentiation. These phenotypes observed in *Taz*- and *Prelid1*-knockdown cells were remarkably similar. These results indicate that the temporal induction of mitochondrial C18:2-containing CL is crucial for the proper progression of myoblast differentiation. Although PRELID1 is known to transport PA for CL synthesis (36), its role in determining the CL acyl chain composition has not been previously reported. Since CL with C18:2 was reduced in both knockdown models and their CL fatty acid profiles were nearly identical, we propose that PRELID1-mediated CL synthesis and Taz-mediated remodeling may be functionally coupled. It has been suggested that Taz acts only on a specific lipid pool, with substrate specificity determined by the physical lipid properties (47). Taz has also been reported to form a part of mitochondrial protein complexes, although the exact components remain unidentified (41). Notably, unlike Taz, Prelid1 expression did not increase with differentiation, raising the possibility that their expression regulation may differ. Future studies should investigate the subcellular localization and functional coupling of Taz and PRELID1.

CL is predominantly localized in the IMM (48) and regulates various bioenergetic processes by interacting with IMM-resident proteins (49, 50, 51). C18:2-enriched CL is the dominant CL species in mammalian tissues (52) and is critical for several mitochondrial functions (39, 53, 54, 55). In support of this, rats fed a C18:2-deficient diet showed decreased levels of C18:2-containing CL in cardiac mitochondria, along with impaired oxygen consumption and cytochrome *c* activity (56). However, the molecular mechanism by which C18:2-containing CL facilitates mitochondrial function remains unclear. Several mitochondrial stressors, including carbonyl cyanide p-(trifluoromethoxy) phenylhydrazone (an uncoupler) (57), rotenone (a complex I inhibitor) (58), and oligomycin A (an ATP synthase inhibitor) (59), inhibit C2C12 myogenic differentiation. We also observed that treatment with oligomycin A inhibited myoblast differentiation. Given that mitochondrial activity increases during myoblast differentiation (43, 44, 60), it is plausible that CL containing C18:2 is required to enhance mitochondrial function, including energy production. We also observed that the increase in CL following C18:2 treatment was significantly suppressed by oligomycin A treatment. Based on these results, we propose a positive feedback loop in which increased C18:2-containing CL supports mitochondrial function, and enhanced mitochondrial activity in turn promotes CL remodeling. A previous study reported that Wnt signaling is downregulated, and Mkx-mediated repression of MyoD is increased in *Taz* KO cells (13). However, our *Taz*- and *Prelid1*-knockdown cells, both of which showed defective myogenic differentiation, did not exhibit significant changes in Wnt signaling (supplemental Figs. S5A and S6A). This suggests that Taz-mediated Wnt signaling regulation may be independent of CL remodeling. Alternatively, severe mitochondrial dysfunction in undifferentiated *Taz*-KO cells (14) may affect Wnt signaling, whereas our knockdown cells retained their functional mitochondrial respiration. Moreover, although it is reported that pyruvate dehydrogenase (PDH) activity is decreased, which is caused by the increase of PDH phosphorylation, in *Taz* KO C2C12 cells (61), *Taz*- or *Prelid1*-knockdown cells did not affect PDH phosphorylation in this study (supplemental Figs. S5B and S6B). These results raise the possibility that acute modification of CL remodeling is sufficient to influence the myoblast differentiation without affecting the basal mitochondrial function in an undifferentiated state. In other words, CL remodeling during myoblast differentiation is important for the proper progress of differentiation.

We observed an increase in CL with C18:2 not only in differentiating C2C12 cells but also in primary cultured murine skeletal MuSCs. Although these primary cells exhibited higher basal levels of C18:2-containing CL, their levels increased further upon differentiation, similar to those in C2C12 cells. This was accompanied by increased Taz protein expression, supporting the notion that lipid remodeling also occurs in vivo. During muscle injury, SCs are activated and undergo differentiation to regenerate muscle fibers (62, 63). To the best of our knowledge, no previous study has examined the mitochondrial lipidome during in vivo muscle regeneration. It would be highly informative to monitor the CL dynamics during this process. Age-related decline in muscle regeneration is well documented (64), and while reduced SC number and function are contributing factors, the underlying mechanisms remain unclear. Recently, we performed a comprehensive lipidomic analysis of mice at various life stages and detected an increase in CL without C18:2 and a decrease in some CL species containing C18:2 in aged skeletal muscles (supplemental Fig. S8C) (38). It has also been reported that cardiac muscles from aged rats show reduced C18:2-containing CL and increased saturated CL species (65). These results suggest that an altered CL acyl composition may contribute to age-related impairments in muscle regeneration. Thus, monitoring and regulating mitochondrial lipid composition could offer a novel diagnostic and/or preventive strategy when dysregulated myogenesis is suspected to be a key component of pathogenesis.

## Data availability

LC-MS/MS data are available at MB-POST (https://repository.massbank.jp/) via the index MPST000052.

## Supplemental data

This article contains supplemental data.

## Conflict of interest

The authors declare that they have no conflicts of interest with the contents of this article.
