# Supplementary material for: Mitochondrial cardiolipin remodeling facilitates efficient myoblast differentiation
Source: J Lipid Res. 2025 Sep 23;66(11):100909. doi: 10.1016/j.jlr.2025.100909 (PMC12605056; doi:10.1016/j.jlr.2025.100909)
Supplement: Supplemental materials [file mmc1.pdf]

Figure S1

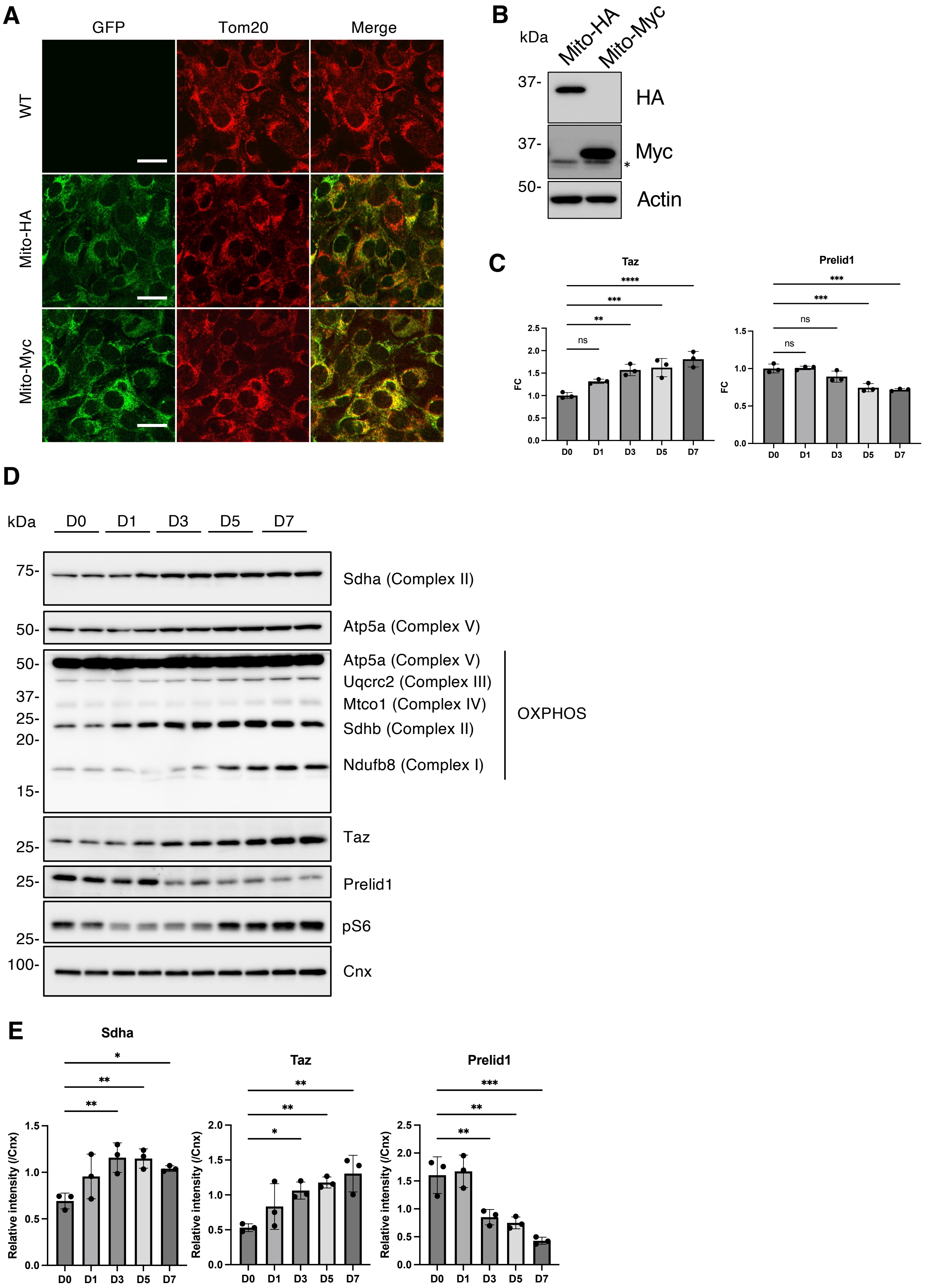
A
GFP
Tom20
Merge
WT
Mito-HA
Mito-Myc
B
kDa
Mito-HA
Mito-Myc
37-
HA
37-
Myc
*
50-
Actin
C
Taz
****
***
**
ns
FC
D0
D1
D3
D5
D7
Prelid1
***
***
ns
ns
FC
D0
D1
D3
D5
D7
D
kDa
D0
D1
D3
D5
D7
75-
Sdha (Complex II)
50-
Atp5a (Complex V)
50-
37-
25-
20-
15-
Atp5a (Complex V)
Uqcrc2 (Complex III)
Mtco1 (Complex IV)
Sdhb (Complex II)
Ndufb8 (Complex I)
OXPHOS
25-
Taz
25-
Prelid1
pS6
25-
100-
Cnx
E
Sdha
*
**
**
Relative intensity (/Cnx)
D0
D1
D3
D5
D7
Taz
**
**
*
Relative intensity (/Cnx)
D0
D1
D3
D5
D7
Prelid1
***
**
**
Relative intensity (/Cnx)
D0
D1
D3
D5
D7

Figure S1
Validation of Mito-tag expression and evaluation of mitochondrial proteins in Mito-HA expressing C2C12 cells

(A) Immunofluorescence of C2C12 cells expressing Mito-HA or Mito-Myc. Cells were immunostained with Tom20 antibodies. Scale bar, 10 μm.

(B) Expression of mitochondrial targeted proteins (Mito-HA or Mito-Myc) were confirmed by immunoblotting. Indicated antibodies were used. * Cross reaction.

(C) The mRNA levels of Taz and Prelid1 during C2C12 cell differentiation were quantified using qRT-PCR (n=3).

(D, E) Immunoblot analysis of mitochondrial proteins during C2C12 cell differentiation (D). Indicated antibodies were used. The protein expression levels of Sdha, Taz and Prelid1 were quantified (E) (n=3).

Mean ± S.D. *P < 0.05, **P < 0.01, ***P < 0.001, ****P < 0.0001, ns; not significant, one-way ANOVA with Dunnett's multiple comparisons test.

Figure S2

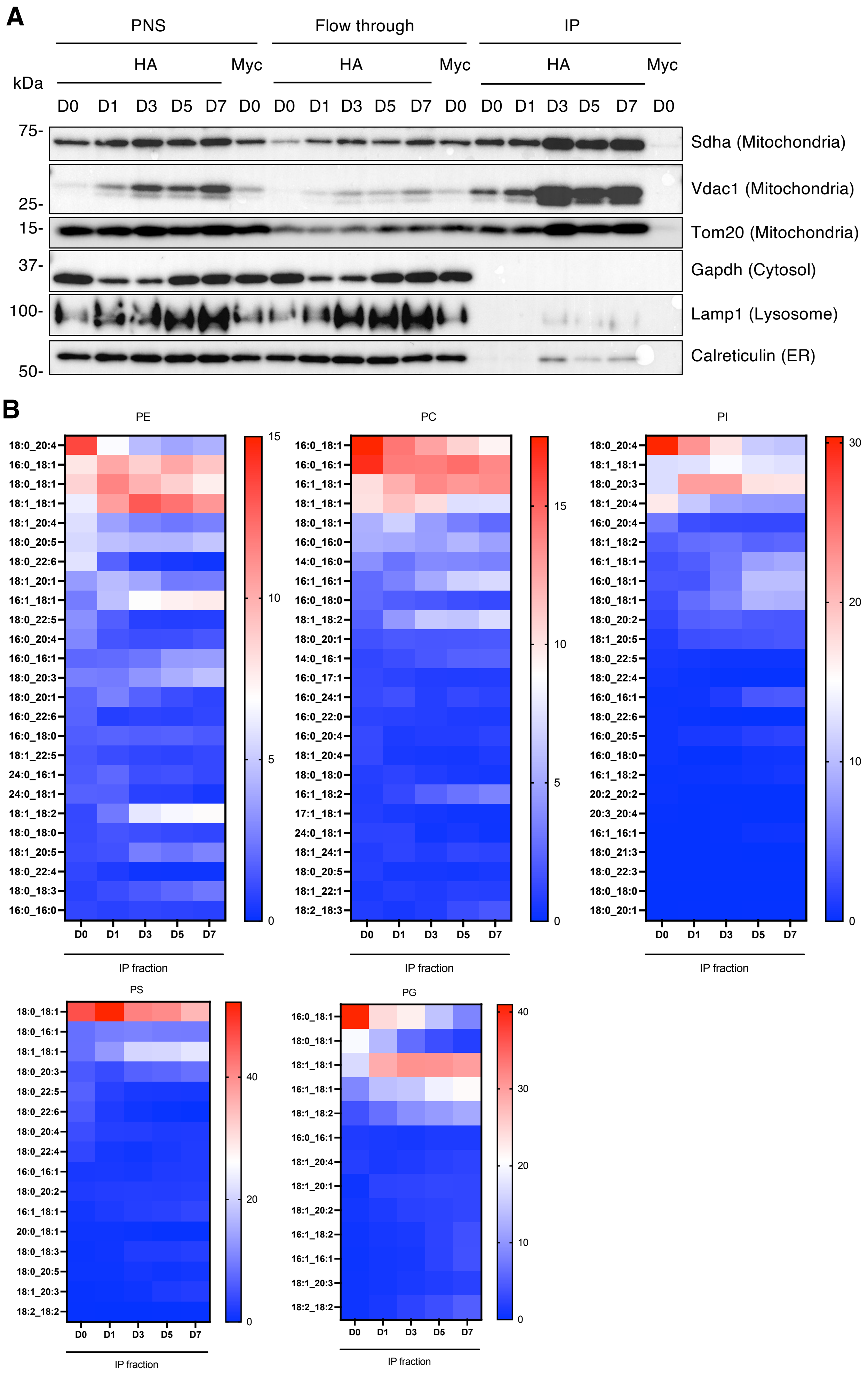
A
PNS
Flow through
IP
HA
Myc
kDa
D0 D1 D3 D5 D7 D0
75-
25-
15-
37-
100-
50-
Sdha (Mitochondria)
Vdac1 (Mitochondria)
Tom20 (Mitochondria)
Gapdh (Cytosol)
Lamp1 (Lysosome)
Calreticulin (ER)
B
PE
18:0_20:4 16:0_18:1 18:0_18:1 18:1_18:1 18:1_20:4 18:0_20:5 18:0_22:6 18:1_20:1 16:1_18:1 18:0_22:5 16:0_20:4 16:0_16:1 18:0_20:3 18:0_20:1 16:0_22:6 16:0_18:0 18:1_22:5 24:0_16:1 24:0_18:1 18:1_18:2 18:0_18:0 18:1_20:5 18:0_22:4 18:0_18:3 16:0_16:0
0 5 10 15
PC
16:0_18:1 16:0_16:1 16:1_18:1 18:1_18:1 18:0_18:1 16:0_16:0 14:0_16:0 16:1_16:1 16:0_18:0 18:1_18:2 18:0_20:1 14:0_16:1 16:0_17:1 16:0_24:1 16:0_22:0 16:0_20:4 18:1_20:4 18:0_18:0 16:1_18:2 17:1_18:1 24:0_18:1 18:1_24:1 18:0_20:5 18:1_22:1 18:2_18:3
0 5 10 15
PI
18:0_20:4 18:1_18:1 18:0_20:3 18:1_20:4 16:0_20:4 18:1_18:2 16:1_18:1 16:0_18:1 18:0_18:1 18:0_20:2 18:1_20:5 18:0_22:5 18:0_22:4 16:0_16:1 18:0_22:6 16:0_20:5 16:0_18:0 16:1_18:2 20:2_20:2 20:3_20:4 16:1_16:1 18:0_21:3 18:0_22:3 18:0_18:0 18:0_20:1
0 10 20 30
PS
18:0_18:1 18:0_16:1 18:1_18:1 18:0_20:3 18:0_22:5 18:0_22:6 18:0_20:4 18:0_22:4 16:0_16:1 18:0_20:2 16:1_18:1 20:0_18:1 18:0_18:3 18:0_20:5 18:1_20:3 18:2_18:2
0 20 40
PG
16:0_18:1 18:0_18:1 18:1_18:1 16:1_18:1 18:1_18:2 16:0_16:1 18:1_20:4 18:1_20:1 18:1_20:2 16:1_18:2 16:1_16:1 18:1_20:3 18:2_18:2
0 10 20 30 40
D0 D1 D3 D5 D7
IP fraction

Figure S2

Mitochondrial membrane phospholipid profiles of Mito-HA C2C12 cells

(A) The enrichment of mitochondria by Mito-IP was confirmed by immunoblotting. Indicated antibodies were used. PNS; post nuclear supernatant, IP; immunoprecipitation, HA; Mito-HA expressing C2C12 cells, Myc; Mito-Myc expressing C2C12 cells, D; days after differentiation induction.

(B) The fatty acid composition of PE, PC, PI, PS, or PG is shown as heatmap with mean value (n=3).

Figure S3

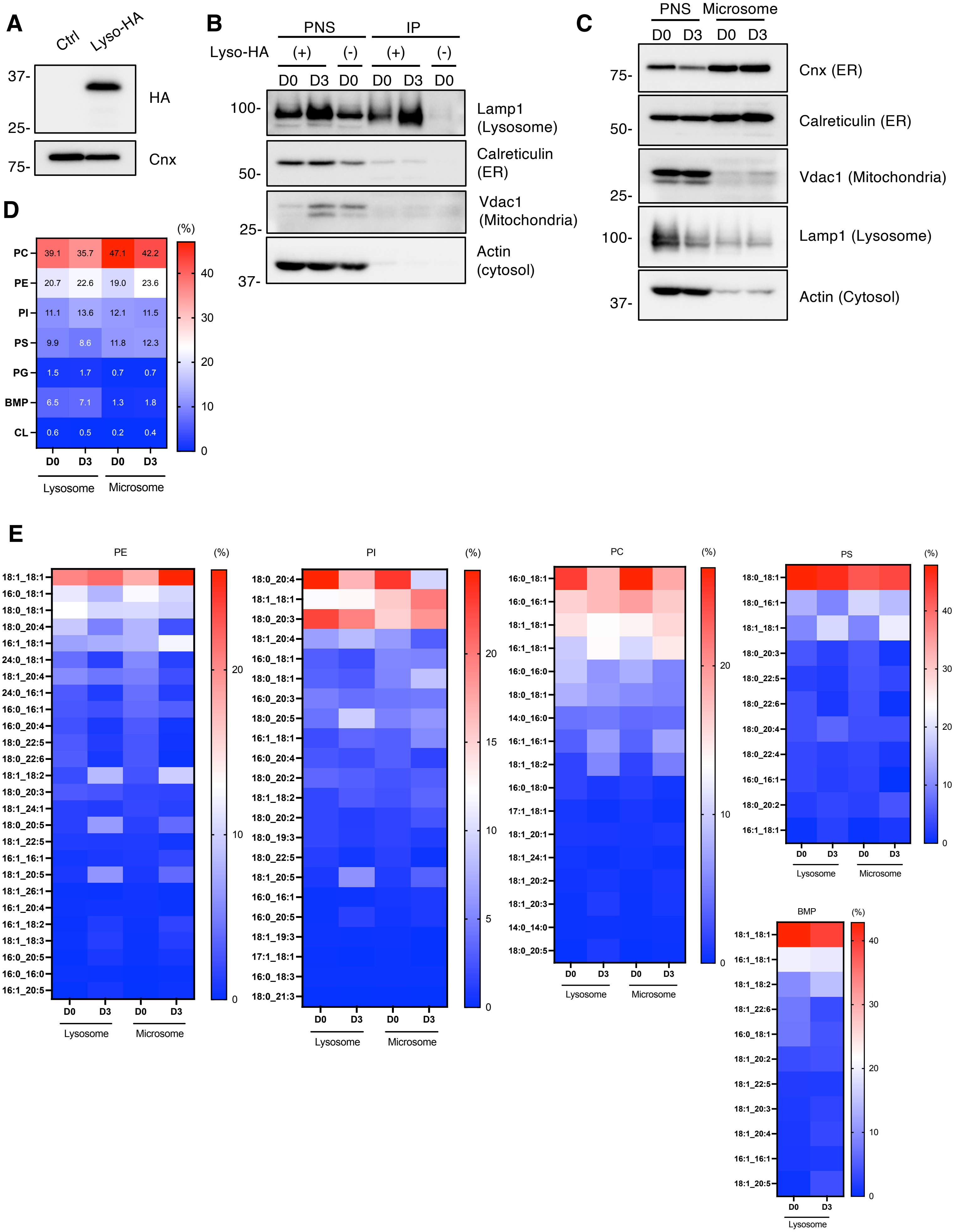

A
Ctrl
Lyso-HA
37-
25-
75-
HA
Cnx
B
PNS
IP
Lyso-HA
(+)
(-)
(+)
(-)
D0
D3
D0
D0
D3
D0
100-
50-
25-
37-
Lamp1 (Lysosome)
Calreticulin (ER)
Vdac1 (Mitochondria)
Actin (cytosol)
C
PNS
Microsome
D0
D3
D0
D3
75-
50-
25-
100-
37-
Cnx (ER)
Calreticulin (ER)
Vdac1 (Mitochondria)
Lamp1 (Lysosome)
Actin (Cytosol)
D
(%)
PC 39.1 35.7 47.1 42.2
PE 20.7 22.6 19.0 23.6
PI 11.1 13.6 12.1 11.5
PS 9.9 8.6 11.8 12.3
PG 1.5 1.7 0.7 0.7
BMP 6.5 7.1 1.3 1.8
CL 0.6 0.5 0.2 0.4
D0
D3
D0
D3
Lysosome
Microsome
40
30
20
10
0
E
PE
PI
PC
PS
BMP
(%)
Lysosome
Microsome

Figure S3

Lysosomal and microsomal membrane phospholipid profiles of C2C12 cells

(A) Expression of lysosomal targeted proteins (Lyso-HA) were confirmed by immunoblotting. Indicated antibodies were used.

(B) The enrichment of lysosome by Lyso-IP was confirmed by immunoblotting. Indicated antibodies were used. PNS; post nuclear supernatant, IP; immunoprecipitation, D; days after differentiation induction.

(C) The isolation of microsomal fraction was confirmed by immunoblotting. Indicated antibodies were used.

(D) The proportion of each phospholipid in either the Lyso-IP fraction or microsomal fraction is shown as a heatmap with mean values (n=3).

(E) The fatty acid composition of PE, PC, PI, PS, or BMP is shown as heatmap with mean value (n=3).

Figure S4

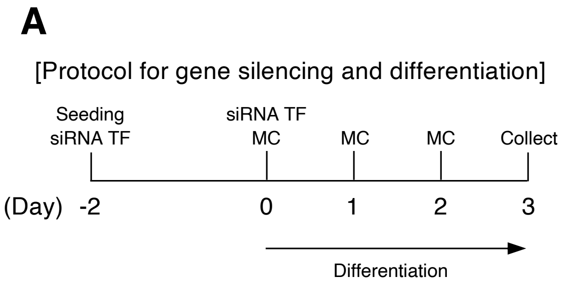

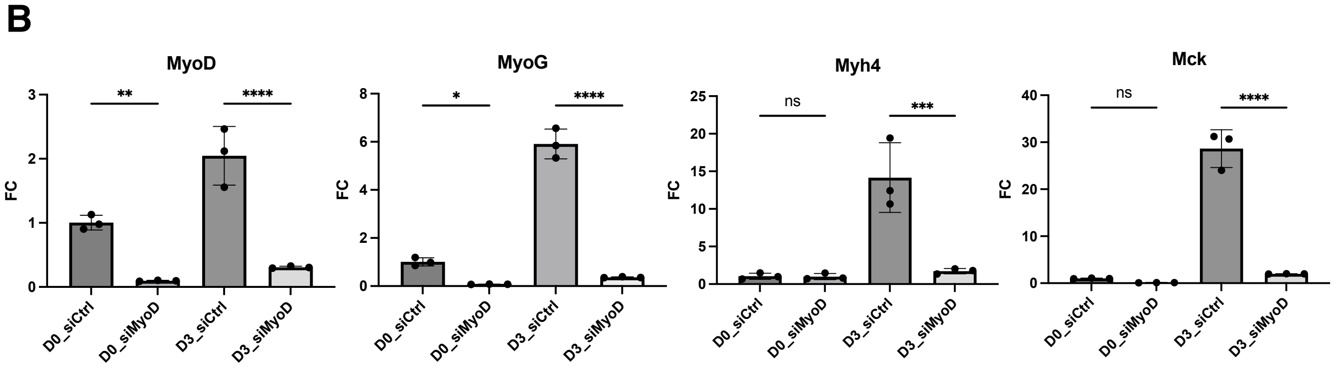

Figure S4

siRNA mediated knockdown of MyoD in C2C12 cells

(A) The scheme of siRNA transfection and myoblast differentiation of C2C12 cells.

(B) The mRNA levels of MyoD, MyoG, Myh4, and Mck during C2C12 cell differentiation were quantified using qRT-PCR (n=3).

Mean ± S.D. *P < 0.05, **P < 0.01, ***P < 0.001, ****P < 0.0001, ns; not significant, one-way ANOVA with Sidak's multiple comparisons test.

Figure S5

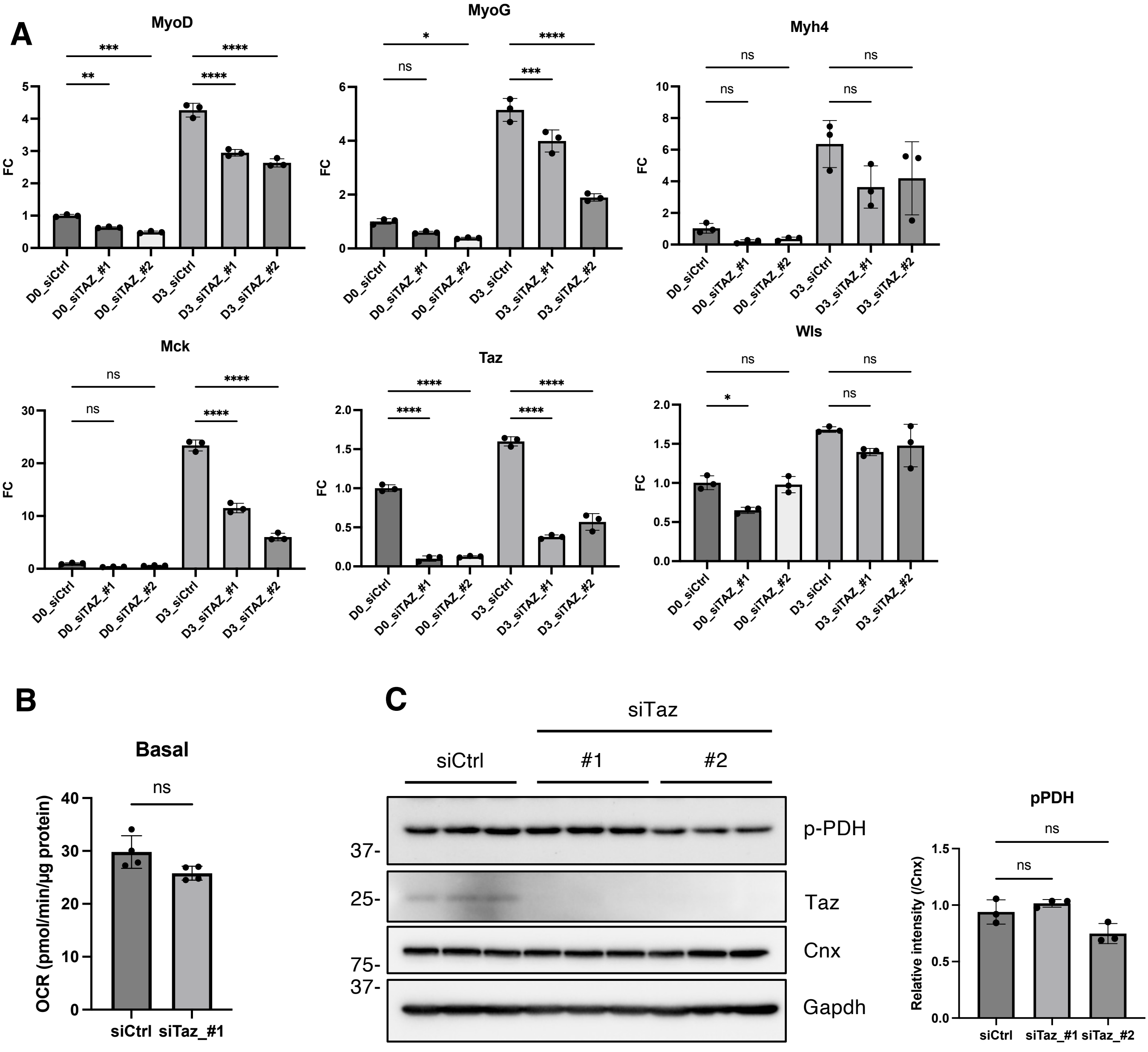

Figure S5

siRNA mediated knockdown of Taz in C2C12 cells

(A) The mRNA levels of indicated genes during myoblast differentiation of either siCtrl or siTaz (#1 or #2) treated C2C12 cells were quantified using qRT-PCR (n=3).

(B) Basal oxygen consumption rate (OCR) of siCtrl or siTaz (#1) treated undifferentiated C2C12 cells were measured using seahorse flux analyzer (n=4).

(C) Immunoblot analysis of siCtrl or siTaz (#1 or #2) treated undifferentiated C2C12 cells. The phosphorylation levels of PDH were quantified (n=3).

Mean ± S.D. *P < 0.05, **P < 0.01, ***P < 0.001, ****P < 0.0001, ns; not significant, one-way ANOVA with Sidak's multiple comparisons test (A), unpaired student's t test (B), or Dunnett's multiple comparisons test (C).

Figure S6

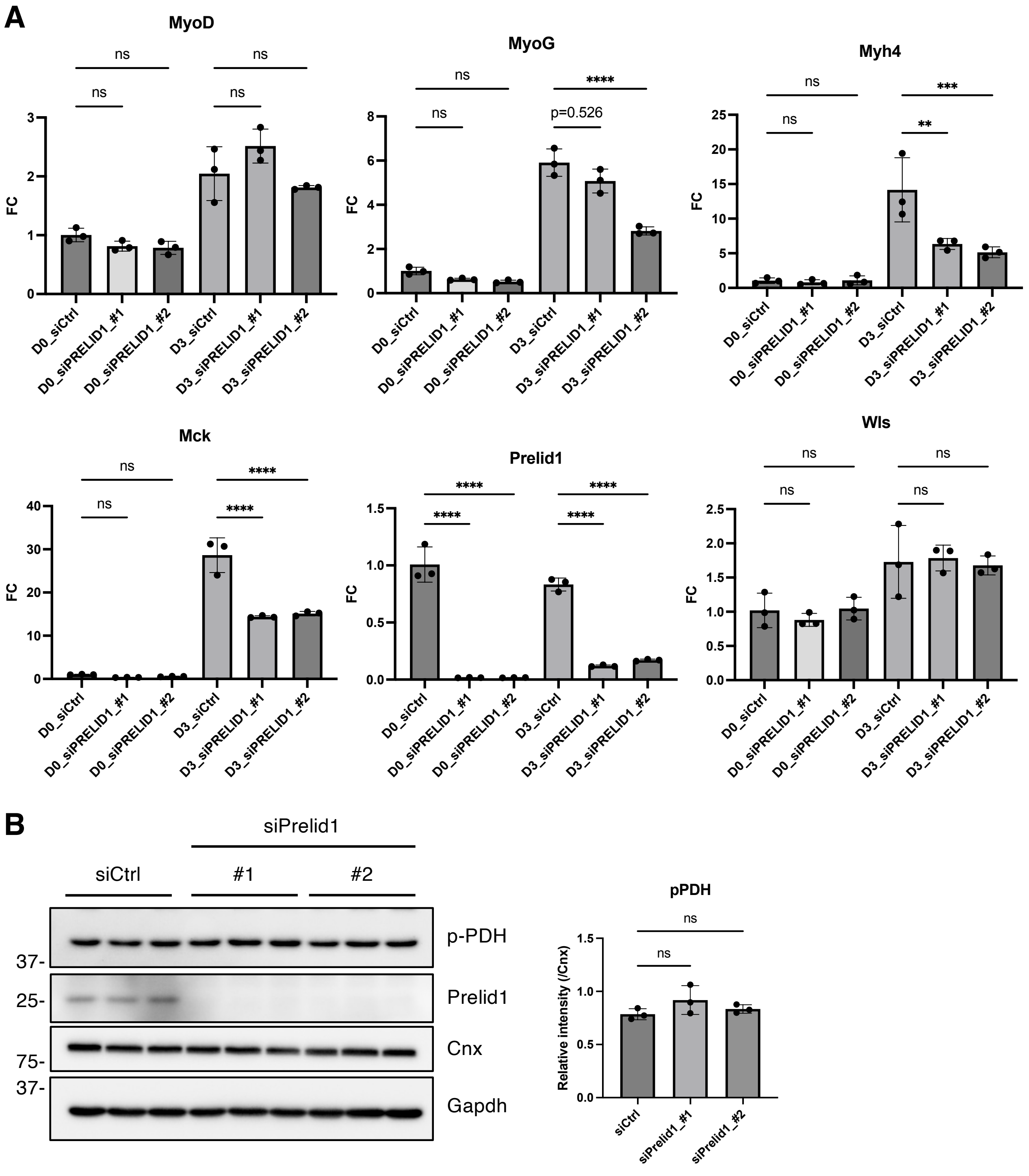

Figure S6

siRNA mediated knockdown of Prelid1 in C2C12 cells

(A) The mRNA levels of indicated genes during myoblast differentiation of either siCtrl or siPrelid1 (#1 or #2) treated C2C12 cells were quantified using qRT-PCR (n=3).

(B) Immunoblot analysis of siCtrl or siPrelid1 (#1 or #2) treated undifferentiated C2C12 cells. The phosphorylation levels of PDH were quantified (n=3).

Mean ± S.D. **P < 0.01, ***P < 0.001, ****P < 0.0001, ns; not significant, one-way ANOVA with Sidak's multiple comparisons test (A), or Dunnett's multiple comparisons test (B).

Figure S7

A

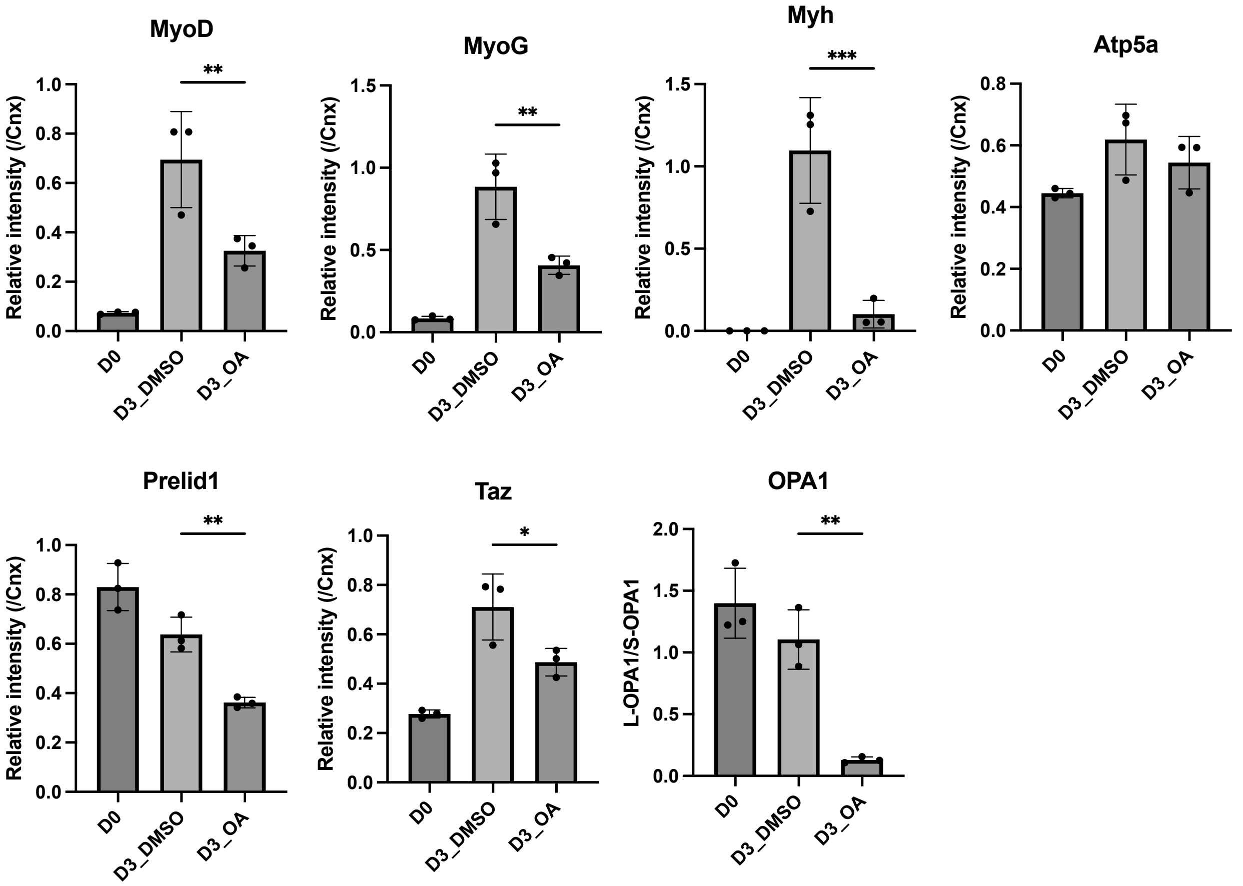

Figure S7

Inhibition of ATP synthase by oligomycin A (OA) in C2C12 cells

(A) The protein expression levels or the ratio of long (L-) and short (S) OPA1 of OA treated (10 nM, 3 days) C2C12 cells evaluated by immunoblot were quantified (B) (n=3). Representative immunoblot data of n=2 out of n=3 is shown in Figure 6B.

Mean ± S.D. *P < 0.05, **P < 0.01, ***P < 0.001, one-way ANOVA with Sidak's multiple comparisons test.

Figure S8

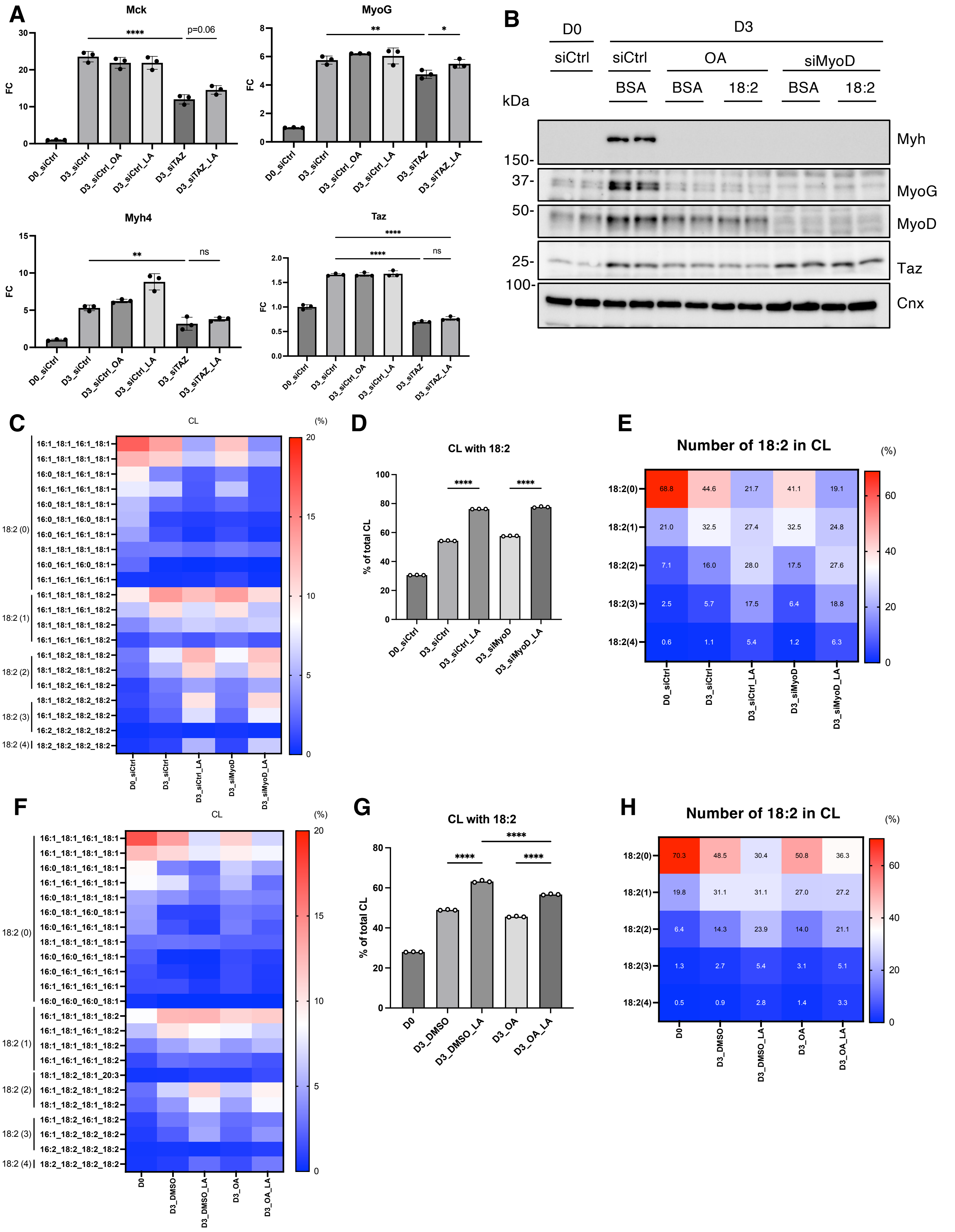

A
Mck
MyoG
Myh4
Taz
FC
****
p=0.06
**
*
ns
D0_siCtrl
D3_siCtrl
D3_siCtrl_OA
D3_siCtrl_LA
D3_siTAZ
D3_siTAZ_LA
B
D0
D3
siCtrl
siCtrl
OA
siMyoD
BSA
BSA
18:2
BSA
18:2
kDa
150-
37-
50-
25-
100-
Myh
MyoG
MyoD
Taz
Cnx
C
CL
(%)
18:2 (0)
18:2 (1)
18:2 (2)
18:2 (3)
18:2 (4)
16:1_18:1_16:1_18:1
16:1_18:1_18:1_18:1
16:0_18:1_16:1_18:1
16:1_16:1_16:1_18:1
16:0_18:1_18:1_18:1
16:0_18:1_16:0_18:1
16:0_16:1_16:1_18:1
18:1_18:1_18:1_18:1
16:0_16:1_16:0_18:1
16:1_16:1_16:1_16:1
16:1_18:1_18:1_18:2
16:1_18:1_16:1_18:2
18:1_18:1_18:1_18:2
16:1_16:1_16:1_18:2
16:1_18:2_18:1_18:2
18:1_18:2_18:1_18:2
16:1_18:2_16:1_18:2
18:1_18:2_18:2_18:2
16:1_18:2_18:2_18:2
16:2_18:2_18:2_18:2
18:2_18:2_18:2_18:2
D0_siCtrl
D3_siCtrl
D3_siCtrl_LA
D3_siMyoD
D3_siMyoD_LA
D
CL with 18:2
% of total CL
****
D0_siCtrl
D3_siCtrl
D3_siCtrl_LA
D3_siMyoD
D3_siMyoD_LA
E
Number of 18:2 in CL
(%)
18:2(0) 68.8 44.6 21.7 41.1 19.1
18:2(1) 21.0 32.5 27.4 32.5 24.8
18:2(2) 7.1 16.0 28.0 17.5 27.6
18:2(3) 2.5 5.7 17.5 6.4 18.8
18:2(4) 0.6 1.1 5.4 1.2 6.3
D0_siCtrl
D3_siCtrl
D3_siCtrl_LA
D3_siMyoD
D3_siMyoD_LA
F
CL
(%)
18:2 (0)
18:2 (1)
18:2 (2)
18:2 (3)
18:2 (4)
16:1_18:1_16:1_18:1
16:1_18:1_18:1_18:1
16:0_18:1_16:1_18:1
16:1_16:1_16:1_18:1
16:0_18:1_18:1_18:1
16:0_18:1_16:0_18:1
16:0_16:1_16:1_18:1
18:1_18:1_18:1_18:1
16:0_16:0_16:1_18:1
16:0_16:1_16:1_16:1
16:1_16:1_16:1_16:1
16:0_16:0_16:0_18:1
16:1_18:1_18:1_18:2
16:1_18:1_16:1_18:2
18:1_18:1_18:1_18:2
16:1_16:1_16:1_18:2
18:1_18:2_18:1_20:3
16:1_18:2_18:1_18:2
18:1_18:2_18:1_18:2
16:1_18:2_16:1_18:2
16:1_18:2_18:2_18:2
16:2_18:2_18:2_18:2
18:2_18:2_18:2_18:2
D0
D3_DMSO
D3_DMSO_LA
D3_OA
D3_OA_LA
G
CL with 18:2
% of total CL
****
D0
D3_DMSO
D3_DMSO_LA
D3_OA
D3_OA_LA
H
Number of 18:2 in CL
(%)
18:2(0) 70.3 48.5 30.4 50.8 36.3
18:2(1) 19.8 31.1 31.1 27.0 27.2
18:2(2) 6.4 14.3 23.9 14.0 21.1
18:2(3) 1.3 2.7 5.4 3.1 5.1
18:2(4) 0.5 0.9 2.8 1.4 3.3
D0
D3_DMSO
D3_DMSO_LA
D3_OA
D3_OA_LA

Figure S8

Linoleic acid supplementation didn't rescue the defect of myoblast differentiation in siMyoD or Oligomycin A (OA) treated C2C12 cells

(A) The mRNA levels of indicated genes during myoblast differentiation of either siCtrl or siTaz (#1) treated C2C12 cells supplemented with oleic acid (18:1) or linoleic acid (18:2) were quantified using qRT-PCR (n=3).

(B) Immunoblot analysis of indicated proteins during myoblast differentiation of either siCtrl, siMyoD or oligomycin A (OA) treated C2C12 cells supplemented with 18:2.

(C, F) The proportion of CL with each fatty acid composition is shown as a heatmap with the mean value (n=3). The number of 18:2 is mentioned on the left side of heatmap.

(D, G) The proportion of CL with 18:2 (n=3).

(E, H) The proportion of CL with each number of 18:2 is shown as a heatmap with the mean value (n=3).

*(C-E) The results of siMyoD treated cells, (F-H) the results of OA treated cells.

Mean ± S.D. **P < 0.01, ****P < 0.0001, ns; not significant, one-way ANOVA with Sidak's multiple comparisons test.

Figure S9

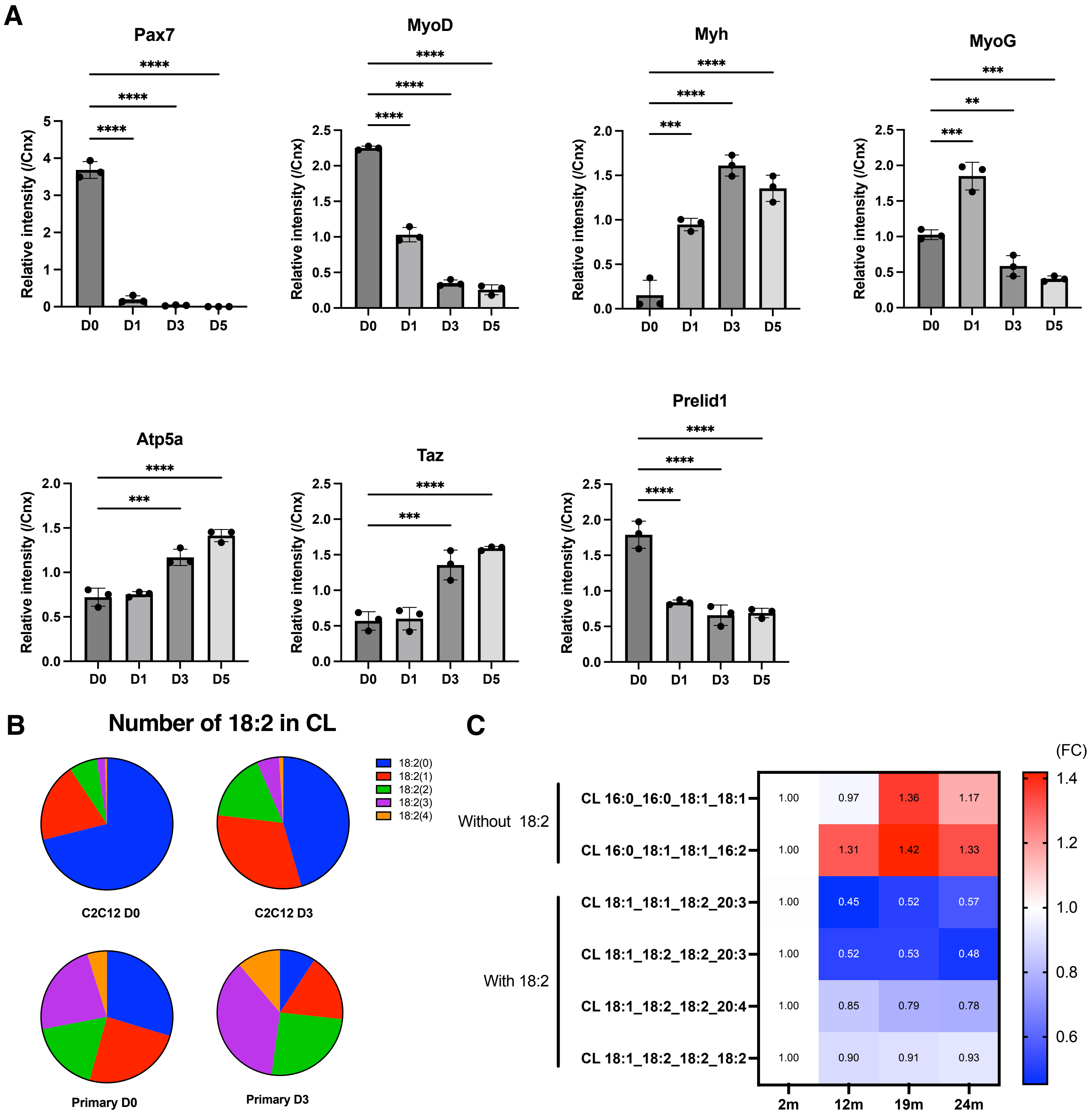

Figure S9

Isolated mouse primary myoblasts were differentiated into myotubes

(A) The protein expression levels of isolated mouse primary myoblasts evaluated by immunoblot were quantified (n=3). Representative immunoblot data of n=2 out of n=3 is shown in Figure 8C.

(B) Comparison of the proportion of CL with different numbers of linoleic acid in primary cultured cells and C2C12 cells. Data are from Figure 3E and Figure 8F.

(C) The transition of the detected CL without C18:2 and the decreased C18:2-containing CL in skeletal muscles is shown as a heatmap (n=3, fold change (FC) vs 2-month-old mice (2m)). Analyzed data are from SPF male mice (2-month-, 12-month-, 19-month-, and 24-month-old) in our previous report (38).

Mean ± S.D. $^{**}P < 0.01$, $^{***}P < 0.001$, $^{****}P < 0.0001$, one-way ANOVA with Dunnett's multiple comparisons test.

Table S1. List of silencer select siRNA

Target gene	silencer select siRNA ID (Thermo)
MyoD	s232596
Taz_#1	s211845
Taz_#2	s83924
Prelid1_#1	s83366
Prelid1_#2	s83367

Table S2. Primers for RT-qPCR

Gene	fw/rv	Sequence (5` to 3')
Mck	fw	CTGACCCCTGACCTCTACAAT
	rv	CATGGCGGTCCTGGATGAT
MyoD	fw	CCACTCCGGGACATAGACTTG
	rv	AAAAGCGCAGGTCTGGTGAG
MyoG	fw	CGGTGGAGGATATGTCTGTTG
	rv	GGTGTTAGCCTTATGTGAATGG
Myh4	fw	CATCTGGTAACACAAGAGGTGC
	rv	GACTTCCGGAGGTAAGGAGC
Taz	fw	CCTGAAGTTGATGCGTTGGA
	rv	GACACACAGGCACACATTTGC
Prelid1	fw	CCATGACCACCTTCACCTGG
	rv	GGGCAAGACCAAATTCCTGG
Wls	fw	ACTGCAGCTTACTACCACTATAAC
	rv	GCCATACCATAGCCTCCTATTC
Actin	fw	GCCAACCGTGAAAAGATGAC
	rv	GAGGCATACAGGGACAGCAC

Table S3 Primary antibody list

Antigen	Vender	Cat#	RRID
Myh	Santacruz	sc-376157	AB_10989398
MyoG	Santacruz	sc-52903	AB_784707
MyoD	Santacruz	sc-377460	AB_2813894
CNX	Abcam	ab22595	AB_2069006
Taz	Santacruz	sc-365810	AB_10842049
Prelid1	Abnova	H00027166-M01	AB_534999
Opa1	BD Biosciences	612606	AB_399888
Atp5a	Abcam	ab14748	AB_301447
Pax7	Santacruz	sc-81648	AB_2159836
Actin	Sigma	A2066	AB_476693
OXPHOS	Abcam	ab110413	AB_2629281
Sdha	Abcam	ab14715	AB_301433
Vdac1	Abcam	ab14734	AB_443084
Tom20	Cell Signaling Technology	42406	AB_2687663
Gapdh	Merck	CB1001	AB_2107426
Lamp1	Cell Signaling Technology	3243	AB_2134478
Calreticulin	Cell Signaling Technology	12238	AB_2688013
HA	MBL	M180-3	AB_10951811
Myc	MBL	M192-3	AB_11160947
pS6	Cell Signaling Technology	2211	AB_331679
pPDH	Millipore	Ap1062	AB_10616069

Created by https://lipidomicstandards.org, version v2.4.0

Contents of Report

Separation Workflow 1
- Overall study design 1
- Lipid extraction 2
- Analytical platform 2
- Quality control 2
- Method qualification and validation 2
- Reporting 3

Sample Descriptions 3
- C2C12cells / Primary myoblasts / Mouse / Cells 3

Lipid Class Descriptions 3
- 1) PC[M+CH3COO]- / Lipid identification 3
- 1) PC[M+CH3COO]- / Lipid quantification 4
- 2) Ceramide non-hydroxy fatty acid-sphingosine (Cer_NS)[M+CH3COO]- / Lipid identification 4
- 2) Ceramide non-hydroxy fatty acid-sphingosine (Cer_NS)[M+CH3COO]- / Lipid quantification 5
- 3) PC O[M+CH3COO]- / Lipid identification 5
- 3) PC O[M+CH3COO]- / Lipid quantification 5
- 4) PE O[M-H]- / Lipid identification 6
- 4) PE O[M-H]- / Lipid quantification 6
- 5) PE[M-H]- / Lipid identification 7
- 5) PE[M-H]- / Lipid quantification 7
- 6) PG[M-H]- / Lipid identification 8
- 6) PG[M-H]- / Lipid quantification 8
- 7) PI[M-H]- / Lipid identification 9
- 7) PI[M-H]- / Lipid quantification 9
- 8) PS[M-H]- / Lipid identification 10
- 8) PS[M-H]- / Lipid quantification 10
- 9) CL[M-H]- / Lipid identification 11
- 9) CL[M-H]- / Lipid quantification 11
- 10) SM[M+CH3COO]- / Lipid identification 12
- 10) SM[M+CH3COO]- / Lipid quantification 12
- 11) FA[M-H]- / Lipid identification 12
- 11) FA[M-H]- / Lipid quantification 13
- 12) DG[M+NH4]+ / Lipid identification 13
- 12) DG[M+NH4]+ / Lipid quantification 13
- 13) BMP[M+NH4]+ / Lipid identification 14
- 13) BMP[M+NH4]+ / Lipid quantification 14
- 14) LPC[M+CH3COO]- / Lipid identification 14
- 14) LPC[M+CH3COO]- / Lipid quantification 15
- 15) LPE[M-H]- / Lipid identification 15
- 15) LPE[M-H]- / Lipid quantification 15
- 16) Ether-linked triacylglycerol (EtherTG)[M+NH4]+ / Lipid identification 16
- 16) Ether-linked triacylglycerol (EtherTG)[M+NH4]+ / Lipid quantification 16
- 17) LPI[M-H]- / Lipid identification 17
- 17) LPI[M-H]- / Lipid quantification 17
- 18) TG[M+NH4]+ / Lipid identification 17
- 18) TG[M+NH4]+ / Lipid quantification 18
- 19) LPS[M-H]- / Lipid identification 18
- 19) LPS[M-H]- / Lipid quantification 18
- 20) LPG[M-H]- / Lipid identification 19
- 20) LPG[M-H]- / Lipid quantification 19

Separation Workflow

Overall study design

Title of the study	Mitochondrial cardiolipin remodeling facilitates efficient myoblast differentiation		
Document creation date	06/04/2025	Corresponding Email	ohba-ys@keio.jp
Principal investigator	Yohsuke Ohba / Makoto Arita	Is the workflow targeted or untargeted?	Untargeted
Institution	Keio University	Clinical	No

Lipid extraction

Extraction method	1-phase system	1-phase system	MeOH:CHCl3:H2O=2:1:0.2
pH adjustment	None	Were internal standards added prior extraction?	Yes

Analytical platform

Ionizaton additives	Ammonium acetate	Resolution at m/z 200 at MS1	35000
Number of separation dimensions	One dimension	Mass accuracy in ppm at MS1	2.5
Separation type 1	LC	Recording mode of raw data at MS1	Centroid mode
Separation mode 1 (liquid)	RP	Mass window for precursor ion isolation (in Da total isolation window)	1
Detector	Mass spectrometer	Mass resolution for detected ion at MS2	High resolution
MS type	QTOF	Resolution at m/z 200 at MS2	25000
MS vendor	Shimadzu	Mass accuracy in ppm at MS2	5
Ion source	ESI	Recording mode of raw data at MS2	Centroid mode
MS Level	MS1, MS2	Was/Were additional dimension/techniques used	No
Mass resolution for detected ion at MS1	High resolution		

Quality control

Blanks	Yes	Quality control	Yes
Type of Blanks	Extraction blank	Type of QC sample	Sample pool

Method qualification and validation

Method validation	Yes	Precision	Yes
Lipid recovery	No	Accuracy	No
Dynamic quantification range	Yes	Guidelines followed	None
Limit of quantitation (LOQ)/Limit of detection (LOD)	Yes		

Reporting

Are reported raw data uploaded into repository?	Yes	Summary data	Quantification and identification data
Link to repository / ID to entry	MB-POST/MPST000052	Raw data upload	Yes
Are metadata available?	Yes		

Sample Descriptions

C2C12cells / Primary myoblasts / Mouse / Cells

Storage and collection conditions	Available	Time to freeze	between 10 and 30 min
Provided preanalytical information	Time to freeze	Snap freezing in liquid N2	Yes
Temperature handling original sample	4-8 °C	Storage temperature	-80 °C
Instant sample preparation	No	Additives	None

Lipid Class Descriptions

1) PC[M+CH3COO]- / Lipid identification

Lipid class	PC	Background check at MS^2	No
MS Level for identification	MS^1, MS^2	Did you presume assumptions for identification?	No
Identification level	Molecular species level	Limit of detection	Signal theshold
MS^1 adduct	[M+CH3COO]-	RT verified by standard	Yes
Isotope correction at MS^1	No	Separation of isobaric/isomeric interferece confirmed	No
MS^2 adduct	[M-H]-	Model for separation prediction	No
Fragments for identification		Lipid Identification Software	MS-DIAL

Fragment name
-(CH3-H)
-FA1(-H)-(CH3-H)
-FA2(-H)-(CH3-H)

Isotope correction at MS^2	No	Data manipulation	Smoothing, Centroiding
MS^1 verified by standard	Yes	Nomenclature for intact lipid molecule	Yes
MS^2 verified by standard	Yes	Nomenclature for fragment ions	No
Background check at MS^1	Yes		

1) PC[M+CH3COO]- / Lipid quantification

Quantitative	Yes	Type I isotope correction	No
MS Level for quantification	MS^1	Limit of quantification	No
Internal lipid standard(s) MS^1		Normalization to reference	No

Internal standard	Endogenous subclass
PC 15:0_18:1(d7)	PC subclass

Type of quantification	Internal standard amount	Lipid Quantification Software	MS-DIAL
Response correction	No	Batch correction	No

2) Ceramide non-hydroxy fatty acid-sphingosine (Cer_NS)[M+CH3COO]- / Lipid identification

Lipid class	Ceramide non-hydroxy fatty acid-sphingosine (Cer_NS)	Background check at MS^2	No
MS Level for identification	MS^1, MS^2	Did you presume assumptions for identification?	No
Identification level	Molecular species level	Limit of detection	Signal theshold
MS^1 adduct	[M+CH3COO]-	RT verified by standard	Yes
Isotope correction at MS^1	No	Separation of isobaric/isomeric interferece confirmed	No
MS^2 adduct	[M-H]-	Model for separation prediction	No
Fragments for identification		Lipid Identification Software	MS-DIAL

Fragment name
Neutral loss of H2O
Neutral loss of CH2O
LCB(-C2H8NO)
FA1(+C2H3N)
Fatty acyl -2H fragment

Isotope correction at MS^2	No	Data manipulation	Smoothing, Centroiding
MS^1 verified by standard	Yes	Nomenclature for intact lipid molecule	Yes
MS^2 verified by standard	Yes	Nomenclature for fragment ions	No
Background check at MS^1	Yes		

2) Ceramide non-hydroxy fatty acid-sphingosine (Cer_NS)[M+CH3COO]- / Lipid quantification

Quantitative	Yes	Type I isotope correction	No
MS Level for quantification	MS^1	Limit of quantification	No
Internal lipid standard(s) MS^1		Normalization to reference	No

Internal standard	Endogenous subclass
Cer 18:1;2O/15:0(d7)	Cer_NS subclass

Type of quantification	Internal standard amount	Lipid Quantification Software	MS-DIAL
Response correction	No	Batch correction	No

3) PC O[M+CH3COO]- / Lipid identification

Lipid class	PC O	Background check at MS^2	No
MS Level for identification	MS^1, MS^2	Did you presume assumptions for identification?	No
Identification level	Molecular species level	Limit of detection	Signal theshold
MS^1 adduct	[M+CH3COO]-	RT verified by standard	Yes
Isotope correction at MS^1	No	Separation of isobaric/isomeric interferece confirmed	No
MS^2 adduct	[M-H]-	Model for separation prediction	No
Fragments for identification		Lipid Identification Software	MS-DIAL

Fragment name
-(CH3-H)
-FA1(-H)-(CH3-H)

Isotope correction at MS^2	No	Data manipulation	Smoothing, Centroiding
MS^1 verified by standard	Yes	Nomenclature for intact lipid molecule	Yes
MS^2 verified by standard	Yes	Nomenclature for fragment ions	No
Background check at MS^1	Yes		

3) PC O[M+CH3COO]- / Lipid quantification

Quantitative	Yes	Type I isotope correction	No
MS Level for quantification	MS^1	Limit of quantification	No
Internal lipid standard(s) MS^1		Normalization to reference	No

Internal standard	Endogenous subclass
PC 15:0_18:1(d7)	EtherPC subclass

Type of quantification	Internal standard amount	Lipid Quantification Software	MS-DIAL
Response correction	No	Batch correction	No

4) PE O[M-H]- / Lipid identification

Lipid class	PE O	Background check at MS^2	No
MS Level for identification	MS^1, MS^2	Did you presume assumptions for identification?	No
Identification level	Molecular species level	Limit of detection	Signal theshold
MS^1 adduct	[M-H]-	RT verified by standard	Yes
Isotope correction at MS^1	No	Separation of isobaric/isomeric interferece confirmed	No
MS^2 adduct	[M-H]-	Model for separation prediction	No
Fragments for identification		Lipid Identification Software	MS-DIAL

Fragment name
Neutral loss of fatty acyl
Fatty acid fragment

Isotope correction at MS^2	No	Data manipulation	Smoothing, Centroiding
MS^1 verified by standard	Yes	Nomenclature for intact lipid molecule	Yes
MS^2 verified by standard	Yes	Nomenclature for fragment ions	No
Background check at MS^1	Yes		

4) PE O[M-H]- / Lipid quantification

Quantitative	Yes	Type I isotope correction	No
MS Level for quantification	MS^1	Limit of quantification	No
Internal lipid standard(s) MS^1		Normalization to reference	No

Internal standard	Endogenous subclass
PE 15:0_18:1(d7)	EtherPE subclass

Type of quantification	Internal standard amount	Lipid Quantification Software	MS-DIAL
Response correction	No	Batch correction	No

5) PE[M-H]- / Lipid identification

Lipid class	PE	Background check at MS^2	No
MS Level for identification	MS^1, MS^2	Did you presume assumptions for identification?	No
Identification level	Molecular species level	Limit of detection	Signal theshold
MS^1 adduct	[M-H]-	RT verified by standard	Yes
Isotope correction at MS^1	No	Separation of isobaric/isomeric interferece confirmed	No
MS^2 adduct	[M-H]-	Model for separation prediction	No
Fragments for identification		Lipid Identification Software	MS-DIAL

Fragment name
HG(PE,196)
-FA1(-H)
-FA2(-H)

Isotope correction at MS^2	No	Data manipulation	Smoothing, Centroiding
MS^1 verified by standard	Yes	Nomenclature for intact lipid molecule	Yes
MS^2 verified by standard	Yes	Nomenclature for fragment ions	No
Background check at MS^1	Yes		

5) PE[M-H]- / Lipid quantification

Quantitative	Yes	Type I isotope correction	No
MS Level for quantification	MS^1	Limit of quantification	No
Internal lipid standard(s) MS^1		Normalization to reference	No

Internal standard	Endogenous subclass
PE 15:0_18:1(d7)	PE subclass

Type of quantification	Internal standard amount	Lipid Quantification Software	MS-DIAL
Response correction	No	Batch correction	No

6) PG[M-H]- / Lipid identification

Lipid class	PG	Background check at MS^2	No
MS Level for identification	MS^1, MS^2	Did you presume assumptions for identification?	No
Identification level	Molecular species level	Limit of detection	Signal theshold
MS^1 adduct	[M-H]-	RT verified by standard	Yes
Isotope correction at MS^1	No	Separation of isobaric/isomeric interferece confirmed	No
MS^2 adduct	[M-H]-	Model for separation prediction	No
Fragments for identification		Lipid Identification Software	MS-DIAL

Fragment name
HG(PG,171)
-FA1(-H)
-FA2(-H)

Isotope correction at MS^2	No	Data manipulation	Smoothing, Centroiding
MS^1 verified by standard	Yes	Nomenclature for intact lipid molecule	Yes
MS^2 verified by standard	Yes	Nomenclature for fragment ions	No
Background check at MS^1	Yes		

6) PG[M-H]- / Lipid quantification

Quantitative	Yes	Type I isotope correction	No
MS Level for quantification	MS^1	Limit of quantification	No
Internal lipid standard(s) MS^1		Normalization to reference	No

Internal standard	Endogenous subclass
PG 15:0_18:1(d7)	PG subclass

Type of quantification	Internal standard amount	Lipid Quantification Software	MS-DIAL
Response correction	No	Batch correction	No

7) PI[M-H]- / Lipid identification

Lipid class	PI	Background check at MS^2	No
MS Level for identification	MS^1, MS^2	Did you presume assumptions for identification?	No
Identification level	Molecular species level	Limit of detection	Signal theshold
MS^1 adduct	[M-H]-	RT verified by standard	Yes
Isotope correction at MS^1	No	Separation of isobaric/isomeric interferece confirmed	No
MS^2 adduct	[M-H]-	Model for separation prediction	No
Fragments for identification		Lipid Identification Software	MS-DIAL
Isotope correction at MS^2	No	Data manipulation	Smoothing, Centroiding
MS^1 verified by standard	Yes	Nomenclature for intact lipid molecule	Yes
MS^2 verified by standard	Yes	Nomenclature for fragment ions	No
Background check at MS^1	Yes		

Fragment name
HG(PI,241)-(H2O)
HG(PI,297)
-FA1(-H)
-FA2(-H)

7) PI[M-H]- / Lipid quantification

Quantitative	Yes	Type I isotope correction	No
MS Level for quantification	MS^1	Limit of quantification	No
Internal lipid standard(s) MS^1		Normalization to reference	No
Type of quantification	Internal standard amount	Lipid Quantification Software	MS-DIAL
Response correction	No	Batch correction	No

Internal standard	Endogenous subclass
PI 15:0_18:1(d7)	PI subclass

8) PS[M-H]- / Lipid identification

Lipid class	PS	Background check at MS^2	No
MS Level for identification	MS^1, MS^2	Did you presume assumptions for identification?	No
Identification level	Molecular species level	Limit of detection	Signal theshold
MS^1 adduct	[M-H]-	RT verified by standard	Yes
Isotope correction at MS^1	No	Separation of isobaric/isomeric interferece confirmed	No
MS^2 adduct	[M-H]-	Model for separation prediction	No
Fragments for identification		Lipid Identification Software	MS-DIAL
Isotope correction at MS^2	No	Data manipulation	Smoothing, Centroiding
MS^1 verified by standard	Yes	Nomenclature for intact lipid molecule	Yes
MS^2 verified by standard	Yes	Nomenclature for fragment ions	No
Background check at MS^1	Yes		

Fragment name
-(C3H5NO2,87)
-FA1(-H)
-FA2(-H)

8) PS[M-H]- / Lipid quantification

Quantitative	Yes	Type I isotope correction	No
MS Level for quantification	MS^1	Limit of quantification	No
Internal lipid standard(s) MS^1		Normalization to reference	No
Type of quantification	Internal standard amount	Lipid Quantification Software	MS-DIAL
Response correction	No	Batch correction	No

Internal standard	Endogenous subclass
PS 15:0_18:1(d7)	PS subclass

9) CL[M-H]- / Lipid identification

Lipid class	CL	Background check at MS^2	No
MS Level for identification	MS^1, MS^2	Did you presume assumptions for identification?	No
Identification level	Molecular species level	Limit of detection	Signal theshold
MS^1 adduct	[M-H]-	RT verified by standard	Yes
Isotope correction at MS^1	No	Separation of isobaric/isomeric interferece confirmed	No
MS^2 adduct	[M-H]-	Model for separation prediction	No
Fragments for identification		Lipid Identification Software	MS-DIAL

Fragment name
Phosphoglycerol - H2O
Fatty acid fragment
Phosphatidic acid

Isotope correction at MS^2	No	Data manipulation	Smoothing, Centroiding
MS^1 verified by standard	Yes	Nomenclature for intact lipid molecule	Yes
MS^2 verified by standard	Yes	Nomenclature for fragment ions	No
Background check at MS^1	Yes		

9) CL[M-H]- / Lipid quantification

Quantitative	Yes	Type I isotope correction	No
MS Level for quantification	MS^1	Limit of quantification	No
Internal lipid standard(s) MS^1		Normalization to reference	No

Internal standard	Endogenous subclass
PG 15:0_18:1(d7)	CL subclass

Type of quantification	Internal standard amount	Lipid Quantification Software	MS-DIAL
Response correction	No	Batch correction	No

10) SM[M+CH3COO]- / Lipid identification

Lipid class	SM	Background check at MS^2	No
MS Level for identification	MS^1, MS^2	Did you presume assumptions for identification?	No
Identification level	Molecular species level	Limit of detection	Signal theshold
MS^1 adduct	[M+CH3COO]-	RT verified by standard	Yes
Isotope correction at MS^1	No	Separation of isobaric/isomeric interferece confirmed	No
MS^2 adduct	[M-H]-	Model for separation prediction	No
Fragments for identification		Lipid Identification Software	MS-DIAL
Isotope correction at MS^2	No	Data manipulation	Smoothing, Centroiding
MS^1 verified by standard	Yes	Nomenclature for intact lipid molecule	Yes
MS^2 verified by standard	Yes	Nomenclature for fragment ions	No
Background check at MS^1	Yes		

Fragment name
-(CH3-H)
HG(PC,168)
Neutral loss of methyl moiety and acyl

10) SM[M+CH3COO]- / Lipid quantification

Quantitative	Yes	Type I isotope correction	No
MS Level for quantification	MS^1	Limit of quantification	No
Internal lipid standard(s) MS^1		Normalization to reference	No
Type of quantification	Internal standard amount	Lipid Quantification Software	MS-DIAL
Response correction	No	Batch correction	No

Internal standard	Endogenous subclass
SM 18:1;2O/18:1(d9)	SM subclass

11) FA[M-H]- / Lipid identification

Lipid class	FA	Limit of detection	Signal theshold
MS Level for identification	MS^1	RT verified by standard	Yes
Identification level	Species level	Separation of isobaric/isomeric interferece confirmed	No
MS^1 adduct	[M-H]-	Model for separation prediction	No
Isotope correction at MS^1	No	Lipid Identification Software	MS-DIAL
MS^1 verified by standard	Yes	Data manipulation	Smoothing, Centroiding
Background check at MS^1	Yes	Nomenclature for intact lipid molecule	Yes
Did you presume assumptions for identification?	No		

11) FA[M-H]- / Lipid quantification

Quantitative	Yes	Type I isotope correction	No
MS Level for quantification	MS^1	Limit of quantification	No
Internal lipid standard(s) MS^1		Normalization to reference	No

Internal standard	Endogenous subclass
LPC 18:1(d7)	FA subclass

Type of quantification	Internal standard amount	Lipid Quantification Software	MS-DIAL
Response correction	No	Batch correction	No

12) DG[M+NH4]+ / Lipid identification

Lipid class	DG	Background check at MS^2	No
MS Level for identification	MS^1, MS^2	Did you presume assumptions for identification?	No
Identification level	Molecular species level	Limit of detection	Signal theshold
MS^1 adduct	[M+NH4]+	RT verified by standard	Yes
Isotope correction at MS^1	No	Separation of isobaric/isomeric interferece confirmed	No
MS^2 adduct	[M+H]+	Model for separation prediction	No
Fragments for identification		Lipid Identification Software	MS-DIAL

Fragment name
Dehydro-monoacyl glycerols
Neutral loss of H2O

Isotope correction at MS^2	No	Data manipulation	Smoothing, Centroiding
MS^1 verified by standard	Yes	Nomenclature for intact lipid molecule	Yes
MS^2 verified by standard	Yes	Nomenclature for fragment ions	No
Background check at MS^1	Yes		

12) DG[M+NH4]+ / Lipid quantification

Quantitative	Yes	Type I isotope correction	No
MS Level for quantification	MS^1	Limit of quantification	No
Internal lipid standard(s) MS^1		Normalization to reference	No

Internal standard	Endogenous subclass
DG 15:0_18:1(d7)	DG subclass

Type of quantification	Internal standard amount	Lipid Quantification Software	MS-DIAL
Response correction	No	Batch correction	No

13) BMP[M+NH4]+ / Lipid identification

Lipid class	BMP	Background check at MS^2	No
MS Level for identification	MS^1, MS^2	Did you presume assumptions for identification?	No
Identification level	Molecular species level	Limit of detection	Signal theshold
MS^1 adduct	[M+NH4]+	RT verified by standard	Yes
Isotope correction at MS^1	No	Separation of isobaric/isomeric interferece confirmed	No
MS^2 adduct	[M+H]+	Model for separation prediction	No
Fragments for identification		Lipid Identification Software	MS-DIAL
Isotope correction at MS^2	No	Data manipulation	Smoothing, Centroiding
MS^1 verified by standard	Yes	Nomenclature for intact lipid molecule	Yes
MS^2 verified by standard	Yes	Nomenclature for fragment ions	No
Background check at MS^1	Yes		

Fragment name
Dehydro-monoacyl glycerols
Neutral loss of glycerophosphate

13) BMP[M+NH4]+ / Lipid quantification

Quantitative	Yes	Type I isotope correction	No
MS Level for quantification	MS^1	Limit of quantification	No
Internal lipid standard(s) MS^1		Normalization to reference	No
Type of quantification	Internal standard amount	Lipid Quantification Software	MS-DIAL
Response correction	No	Batch correction	No

Internal standard	Endogenous subclass
PG 15:0_18:1(d7)	BMP subclass

14) LPC[M+CH3COO]- / Lipid identification

Lipid class	LPC	Background check at MS^2	No
MS Level for identification	MS^1, MS^2	Did you presume assumptions for identification?	No
Identification level	Molecular species level	Limit of detection	Signal theshold
MS^1 adduct	[M+CH3COO]-	RT verified by standard	Yes
Isotope correction at MS^1	No	Separation of isobaric/isomeric interferece confirmed	No
MS^2 adduct	[M-H]-	Model for separation prediction	No
Fragments for identification		Lipid Identification Software	MS-DIAL
Isotope correction at MS^2	No	Data manipulation	Smoothing, Centroiding
MS^1 verified by standard	Yes	Nomenclature for intact lipid molecule	Yes
MS^2 verified by standard	Yes	Nomenclature for fragment ions	No
Background check at MS^1	Yes		

Fragment name
-(CH3-H)
Fatty acid fragment

14) LPC[M+CH3COO]- / Lipid quantification

Quantitative	Yes	Type I isotope correction	No
MS Level for quantification	MS^1	Limit of quantification	No
Internal lipid standard(s) MS^1		Normalization to reference	No

Internal standard	Endogenous subclass
LPC 18:1 (d7)	LPC subclass

Type of quantification	Internal standard amount	Lipid Quantification Software	MS-DIAL
Response correction	No	Batch correction	No

15) LPE[M-H]- / Lipid identification

Lipid class	LPE	Background check at MS^2	No
MS Level for identification	MS^1, MS^2	Did you presume assumptions for identification?	No
Identification level	Molecular species level	Limit of detection	Signal theshold
MS^1 adduct	[M-H]-	RT verified by standard	Yes
Isotope correction at MS^1	No	Separation of isobaric/isomeric interferece confirmed	No
MS^2 adduct	[M-H]-	Model for separation prediction	No
Fragments for identification		Lipid Identification Software	MS-DIAL

Fragment name
-FA1(-H)
Characteristicfragment(C5H11NO5P-)

Isotope correction at MS^2	No	Data manipulation	Smoothing, Centroiding
MS^1 verified by standard	Yes	Nomenclature for intact lipid molecule	Yes
MS^2 verified by standard	Yes	Nomenclature for fragment ions	No
Background check at MS^1	Yes		

15) LPE[M-H]- / Lipid quantification

Quantitative	Yes	Type I isotope correction	No
MS Level for quantification	MS^1	Limit of quantification	No
Internal lipid standard(s) MS^1		Normalization to reference	No

Internal standard	Endogenous subclass
LPE 18:1 (d7)	LPE subclass

Type of quantification	Internal standard amount	Lipid Quantification Software	MS-DIAL
Response correction	No	Batch correction	No

16) Ether-linked triacylglycerol (EtherTG)[M+NH4]+ / Lipid identification

Lipid class	Ether-linked triacylglycerol (EtherTG)	Background check at MS^2	No
MS Level for identification	MS^1, MS^2	Did you presume assumptions for identification?	No
Identification level	Molecular species level	Limit of detection	Signal theshold
MS^1 adduct	[M+NH4]+	RT verified by standard	Yes
Isotope correction at MS^1	No	Separation of isobaric/isomeric interferece confirmed	No
MS^2 adduct	[M+H]+	Model for separation prediction	No
Fragments for identification		Lipid Identification Software	MS-DIAL
Isotope correction at MS^2	No	Data manipulation	Smoothing, Centroiding
MS^1 verified by standard	Yes	Nomenclature for intact lipid molecule	Yes
MS^2 verified by standard	Yes	Nomenclature for fragment ions	No
Background check at MS^1	Yes		

Fragment name
Neutral loss of fatty acyl and H2O
Neutral loss of alkyl ether

16) Ether-linked triacylglycerol (EtherTG)[M+NH4]+ / Lipid quantification

Quantitative	Yes	Type I isotope correction	No
MS Level for quantification	MS^1	Limit of quantification	No
Internal lipid standard(s) MS^1		Normalization to reference	No
Type of quantification	Internal standard amount	Lipid Quantification Software	MS-DIAL
Response correction	No	Batch correction	No

Internal standard	Endogenous subclass
TG 15:0_18:1(d7)_15:0	EtherTG subclass

17) LPI[M-H]- / Lipid identification

Lipid class	LPI	Background check at MS^2	No
MS Level for identification	MS^1, MS^2	Did you presume assumptions for identification?	No
Identification level	Molecular species level	Limit of detection	Signal theshold
MS^1 adduct	[M-H]-	RT verified by standard	Yes
Isotope correction at MS^1	No	Separation of isobaric/isomeric interferece confirmed	No
MS^2 adduct	[M-H]-	Model for separation prediction	No
Fragments for identification		Lipid Identification Software	MS-DIAL

Fragment name
Phosphoinositol -H2O fragment
Characteristic fragment (C9H16O10P-)
Fatty acid fragment

Isotope correction at MS^2	No	Data manipulation	Smoothing, Centroiding
MS^1 verified by standard	Yes	Nomenclature for intact lipid molecule	Yes
MS^2 verified by standard	Yes	Nomenclature for fragment ions	No
Background check at MS^1	Yes		

17) LPI[M-H]- / Lipid quantification

Quantitative	Yes	Type I isotope correction	No
MS Level for quantification	MS^1	Limit of quantification	No
Internal lipid standard(s) MS^1		Normalization to reference	No

Internal standard	Endogenous subclass
LPC 18:1 (d7)	LPI subclass

Type of quantification	Internal standard amount	Lipid Quantification Software	MS-DIAL
Response correction	No	Batch correction	No

18) TG[M+NH4]+ / Lipid identification

Lipid class	TG	Background check at MS^2	No
MS Level for identification	MS^1, MS^2	Did you presume assumptions for identification?	No
Identification level	Molecular species level	Limit of detection	Signal theshold
MS^1 adduct	[M+NH4]+	RT verified by standard	Yes
Isotope correction at MS^1	No	Separation of isobaric/isomeric interferece confirmed	No
MS^2 adduct	[M+H]+	Model for separation prediction	No
Fragments for identification		Lipid Identification Software	MS-DIAL

Fragment name
Neutral loss of fatty acyl and H2O

Isotope correction at MS^2	No	Data manipulation	Smoothing, Centroiding
MS^1 verified by standard	Yes	Nomenclature for intact lipid molecule	Yes
MS^2 verified by standard	Yes	Nomenclature for fragment ions	No
Background check at MS^1	Yes		

18) TG[M+NH4]+ / Lipid quantification

Quantitative	Yes	Type I isotope correction	No
MS Level for quantification	MS^1	Limit of quantification	No
Internal lipid standard(s) MS^1		Normalization to reference	No

Internal standard	Endogenous subclass
TG 15:0_18:1(d7)_15:0	TG subclass

Type of quantification	Internal standard amount	Lipid Quantification Software	MS-DIAL
Response correction	No	Batch correction	No

19) LPS[M-H]- / Lipid identification

Lipid class	LPS	Background check at MS^2	No
MS Level for identification	MS^1, MS^2	Did you presume assumptions for identification?	No
Identification level	Molecular species level	Limit of detection	Signal theshold
MS^1 adduct	[M-H]-	RT verified by standard	Yes
Isotope correction at MS^1	No	Separation of isobaric/isomeric interferece confirmed	No
MS^2 adduct	[M-H]-	Model for separation prediction	No
Fragments for identification		Lipid Identification Software	MS-DIAL

Fragment name
Neutral loss of C3H6NO2
Phosphoglycerol -H2O fragment

Isotope correction at MS^2	No	Data manipulation	Smoothing, Centroiding
MS^1 verified by standard	Yes	Nomenclature for intact lipid molecule	Yes
MS^2 verified by standard	Yes	Nomenclature for fragment ions	No
Background check at MS^1	Yes		

19) LPS[M-H]- / Lipid quantification

Quantitative	Yes	Type I isotope correction	No
MS Level for quantification	MS^1	Limit of quantification	No
Internal lipid standard(s) MS^1		Normalization to reference	No

Internal standard	Endogenous subclass
LPC 18:1 (d7)	LPS subclass

Type of quantification	Internal standard amount	Lipid Quantification Software	MS-DIAL
Response correction	No	Batch correction	No

20) LPG[M-H]- / Lipid identification

Lipid class	LPG	Background check at MS^2	No
MS Level for identification	MS^1, MS^2	Did you presume assumptions for identification?	No
Identification level	Molecular species level	Limit of detection	Signal theshold
MS^1 adduct	[M-H]-	RT verified by standard	Yes
Isotope correction at MS^1	No	Separation of isobaric/isomeric interferece confirmed	No
MS^2 adduct	[M-H]-	Model for separation prediction	No
Fragments for identification		Lipid Identification Software	MS-DIAL
Isotope correction at MS^2	No	Data manipulation	Smoothing, Centroiding
MS^1 verified by standard	Yes	Nomenclature for intact lipid molecule	Yes
MS^2 verified by standard	Yes	Nomenclature for fragment ions	No
Background check at MS^1	Yes		

Fragment name
Phosphoglycerol -H2O fragment

20) LPG[M-H]- / Lipid quantification

Quantitative	Yes	Type I isotope correction	No
MS Level for quantification	MS^1	Limit of quantification	No
Internal lipid standard(s) MS^1		Normalization to reference	No
Type of quantification	Internal standard amount	Lipid Quantification Software	MS-DIAL
Response correction	No	Batch correction	No

Internal standard	Endogenous subclass
LPC 18:1 (d7)	LPG subclass
